# CD4^+^ and CD8a^+^ PET imaging predicts response to novel PD-1 checkpoint inhibitor: studies of Sym021 in syngeneic mouse cancer models

**DOI:** 10.7150/thno.37513

**Published:** 2019-10-18

**Authors:** Lotte K. Kristensen, Camilla Fröhlich, Camilla Christensen, Maria C. Melander, Thomas T. Poulsen, Gunther R. Galler, Johan Lantto, Ivan D. Horak, Michael Kragh, Carsten H. Nielsen, Andreas Kjaer

**Affiliations:** 1 Minerva Imaging, Copenhagen, Denmark; 2 Dept. of Clinical Physiology, Nuclear Medicine & PET and Cluster for Molecular Imaging, Dept. of Biomedical Sciences, Rigshospitalet and University of Copenhagen, Denmark; 3 Symphogen A/S, Ballerup, Denmark

**Keywords:** Molecular imaging, positron emission tomography (PET), immune cell imaging, immunotherapy, immune checkpoint inhibition, PD-1, lymphocytes, tumor infiltrating lymphocytes, T-cells, CD4, CD8.

## Abstract

Predicting the outcome of immunotherapy is essential for efficient treatment. The recent clinical success of immunotherapy is increasingly changing the paradigm of cancer treatment. Accordingly, the development of immune-based agents is accelerating and the number of agents in the global immuno-oncology pipeline has grown 60-70% over the past year. However, despite remarkable clinical efficacy in some patients, only few achieve a lasting clinical response. Treatment failure can be attributed to poorly immunogenic tumors that do not attract tumor infiltrating lymphocytes (TILs). Therefore, we developed positron emission tomography (PET) radiotracers for non-invasive detection of CD4^+^ and CD8a^+^ TILs in syngeneic mouse tumor models for preclinical studies.

**Methods:** Seven syngeneic mouse tumor models (B16F10, P815, CT26, MC38, Renca, 4T1, Sa1N) were quantified for CD4^+^ and CD8a^+^ TILs using flow cytometry and immunohistochemistry (IHC), as well as for tumor growth response to Sym021, a humanized PD-1 antibody cross-reactive with mouse PD-1**.** Radiotracers were generated from F(ab)'2 fragments of rat-anti-mouse CD4 and CD8a antibodies conjugated to the *p*-SCN-Bn-Desferrioxamine (SCN-Bn-DFO) chelator and radiolabeled with Zirconium-89 (^89^Zr-DFO-CD4/^89^Zr-DFO-CD8a). Tracers were optimized for *in vivo* PET/CT imaging in CT26 tumor-bearing mice and specificity was evaluated by depletion studies and isotype control imaging. ^89^Zr-DFO-CD4 and ^89^Zr-DFO-CD8a PET/CT imaging was conducted in the panel of syngeneic mouse models prior to immunotherapy with Sym021.

**Results:** Syngeneic tumor models were characterized as “hot” or “cold” according to number of TILs determined by flow cytometry and IHC. ^89^Zr-DFO-CD4 and ^89^Zr-DFO-CD8a were successfully generated with a radiochemical purity >99% and immunoreactivity >85%. The optimal imaging time-point was 24 hours post-injection of ~1 MBq tracer with 30 µg non-labeled co-dose. Reduced tumor and spleen uptake of ^89^Zr-DFO-CD8a was observed in CD8a^+^ depleted mice and the uptake was comparable with that of isotype control (^89^Zr-DFO-IgG2b) confirming specificity. PET imaging in syngeneic tumor models revealed a varying maximum tumor-to-heart ratio of ^89^Zr-DFO-CD4 and ^89^Zr-DFO-CD8a across tumor types and in-between subjects that correlated with individual response to Sym021 at day 10 relative to start of therapy (*p=0.0002* and *p=0.0354*, respectively). The maximum ^89^Zr-DFO-CD4 tumor-to-heart ratio could be used to stratify mice according to Sym021 therapy response and overall survival was improved in mice with a ^89^Zr-DFO-CD4 ratio >9 (*p=0.0018*).

Conclusion: We developed ^89^Zr-DFO-CD4 and ^89^Zr-DFO-CD8a PET radiotracers for specific detection and whole-body assessment of CD4^+^ and CD8a^+^ status. These radiotracers can be used to phenotype preclinical syngeneic mouse tumor models and to predict response to an immune checkpoint inhibitor. We foresee development of such non-invasive *in vivo* biomarkers for prediction and evaluation of clinical efficacy of immunotherapeutic agents, such as Sym021.

## Introduction

The rapidly evolving field of cancer immunotherapy and the clinical success of immune checkpoint inhibitors have boosted the development of immune-based agents and changed the standard of care for many types of cancer 1,2. Despite remarkable clinical efficacy in some patients, others fail to develop a durable clinical response. The fact that only a subpopulation of patients benefits from immune checkpoint inhibitors 3 highlights the complexity of the immuno-oncology landscape and the strong urgency to develop improved methods to predict and monitor therapeutic responses of such agents.

The level of immune infiltration, i.e. immune status, in tumors can strongly influence patient outcomes for immune checkpoint inhibitors. Treatment failure can be attributed to the poorly immunogenic, so-called “cold” tumors that do not attract tumor infiltrating lymphocytes (TILs). In particular, preclinical and clinical research has focused on targeting tumors by enhancing cytotoxic CD8^+^ effector function. Indeed, the major players exerting tumor-directed killing are thought to be the CD8^+^ T cells which can be either resident or attracted to the inflamed tumor microenvironment upon presentation of tumor-antigen on MHC class I molecules 4,5. Since most tumors do not express MHC class II molecules, the role of CD4^+^ T cells have been indecipherable. However, several characteristics of CD4^+^ T cells place them as required players for efficacious anti-tumor immunity. CD4^+^ T cells orchestrate a broad range of immune responses including initiating, maintaining and integrating adaptive and innate effector functions. Further, studies in mice have shown that CD4^+^ T cells are required for inducing CD8^+^ anti-tumor responses 6,7. Lastly, CD4^+^ T cells can target tumor cells indirectly by modulating the tumor microenvironment or directly by cytolytic mechanisms 7-10. Not surprisingly, increasing evidence suggests that the presence, location and density of both CD4^+^ and CD8^+^ lymphocytes in the tumor microenvironment of patients are primary drivers for productive anti-tumor immune responses and are predictors of overall survival 11-16.

Despite the association of TIL location and density in the tumor microenvironment with prognosis in various cancers, validated and standardized companion diagnostics assays are still lacking. Common approaches to detect and monitor immune responses are limited to peripheral blood lymphocyte analysis and tumor biopsies that are invasive, prone to sampling error and may not reflect the spatio-temporal dynamics of TILs within the tumor. In contrast, non-invasive molecular imaging with positron emission tomography (PET) allows for a much more comprehensive look at the entire tumor and prospective metastases *in vivo* over time. The ability to monitor TILs over the course of therapy with PET may allow for early determination of treatment efficacy and has thus fueled the development of T cell specific PET probes targeting a variety of surface markers such as PD-1 17-19, CTLA-4 20, CD3^+^ 21,22, CD4^+^ 23 and CD8^+^ 24,25 for the purpose of detection and monitoring of responses to immunotherapy. One key question is however, whether these probes can predict the outcome of checkpoint blockade therapy. To our knowledge, no studies have investigated the predictive value of T cell specific imaging and immune phenotyping prior to immunotherapy. Thus, we sought to develop specific PET radiotracers for non-invasive *in vivo* detection and quantification of TILs in a panel of commonly used preclinical syngeneic mouse models mimicking a broad patient population prior to immune checkpoint inhibition.

In the present study, we utilize the high specificity of antibodies and produce F(ab)'2 fragments towards CD4 and CD8a surface markers. We radiolabel the F(ab)'2 fragments with Zirconium-89 (^89^Zr, t_1/2_=78.4 hours), an isotope well-matched to the biological half-life of F(ab)'2 fragments and validate the specificity of these antibody-based radiotracers for immune phenotyping of tumors. Furthermore, we demonstrate that tumor uptake of CD4^+^ and CD8a^+^ specific tracers is overall associated with the tumor growth response to Sym021. Sym021 is a recombinant, fully human, IgG1-LALA antibody derived from chicken that binds human PD-1 with nanomolar affinity and cross-reacts with mouse PD-1 with a stability similar to fully human antibodies in clinical development 26. Lastly, we show that ^89^Zr-DFO-CD4 can be used to stratify mice into responders and non-responders.

## Materials and methods

### Cell culture and animal models

Murine cancer cell lines (B16F10 (skin, CRL-6475), P815 (mast cell, TIB-64), CT26 (colon, CRL-2638), Renca (kidney, CRL-2947), and 4T1 (breast, CRL-2539)) were purchased from the American Type Culture Collection. Murine cancer cell lines (Sa1N (fibroblast) and MC38 (colon)) were a kind gift from Holbrook Kohrt, Stanford University. The CT26, MC38, 4T1, Renca and Sa1N cells were cultured in RPMI-1640+Glutamax, 10% fetal bovine serum (FBS), 1% penicillin-streptomycin (PS), and the Renca cell line was supplemented with 10 mM HEPES, 2 mM sodium pyruvate and 0.1 mM NEAA. The B16F10 and P815 cells were cultured in DMEM+Glutamax, 10% FBS, 1% PS. P815 was supplemented with 1 mM sodium pyruvate.

All cell lines were maintained at 37°C in a humidified incubator containing 5% CO_2_. Cells were harvested in their exponential growth phase and resuspended in complete growth media at a concentration of 10x10^6^ cells/mL. Cells (100 µL, 1x10^6^ cells) were subcutaneously injected into the right flanks above the hindlimbs in 7-8 week old female mice: C57BL/6 (MC38 and B16F10), BALB/c (CT26, Renca, and 4T1), A/J (Sa1N), and DBA/2 (P815). C57BL/6 and BALB/c mice were supplied by Janvier Labs (France), A/J mice by Envigo (Germany) and DBA/2 mice by Charles River (Germany) and were acclimatized for 1 week prior to experimentation.

Tumor volume was measured by caliper by using the formula (width^2^×length)×0.52. All animal procedures were conducted under a protocol approved by the Danish National Animal Experiments Inspectorate.

### Flow cytometry

Tumors were harvested at an average size of 150-200 mm^3^ and processed into single cell suspensions using a tumor cell isolation kit and the GentleMACS Octo Dissociator (Miltenyi Biotec) according to the manufacturer's descriptions. One million cells were pre-incubated with Fc block (#553141, BD Pharmingen) washed and stained for cell surface markers according to standard procedures. The following mouse antibodies were used: anti-CD8a (PerCP Cy5.5, clone 53-7.6, #100734, BioLegend) and anti-CD4 (FITC, clone RM4-5, #553046, from BD Pharmingen). Cells were acquired on a FACSVerse flow cytometer (BD Biosciences). Data were collected using BD FACSuite Software (v1.6) and further analyzed with FlowJo v10.4.2 (Tree Star Inc.).

### Immunohistochemistry and hematoxylin & eosin staining

Tumors were harvested at an average tumor size of 150-200 mm^3^ and snap frozen. Tissues were cryosectioned at 8 µm and immunohistochemical (IHC) experiments were conducted as follows: slides were fixed in cold acetone or 4% formalin, blocked in 5% normal mouse serum (Jackson Immunoresearch) and incubated with primary antibodies for 1.5 h at room temperature. Primary antibodies were anti-CD3 (Dako, A0452, #280), anti-CD4 (Affymetrix, #14-0042) and anti-CD8a (Affymetrix, #14-0081). Sections were dried and stained with haematoxylin/eosin in a Leica ST4040 automatic stainer.

Assessment of T cell infiltration was performed in a blinded fashion and scored as 0 (negative), 1 (<150 cells per mm^2^), 1-2 (150-300 cells per mm^2^), 2 (300-500 cells per mm^2^, 2-3 (500-800 cells per mm^2^), and 3 (>800 cells per mm^2)^. N=3 per syngeneic tumor model.

### Generation of F(ab)'2 fragments, conjugation and radiolabelling

F(ab)'2 fragments were generated from rat-anti-mouse CD4 clone CK 1.5 (#BE0003-1, BioXcell), rat-anti-mouse CD8a clone YTS169.4 (#BE0117, BioXcell) and rat-anti-mouse IgG2b clone LTF-2 (#BE0090, BioXcell) using FabRICATOR (#A0-FR1-050, Genovis, Sweden). 2.5 mg of antibody in PBS was incubated with 500 units FabRICATOR for 2.5 hours at 37 °C under continuous rotation. The antibody-enzyme mixture was purified by preparative HPLC (Yarra-2000 SEC column, 0.1 M phosphate buffer, 1 mL/min), yielding isolated F(ab)'2 and Fc fragments. F(ab)'2 fragments were randomly conjugated to *p*-SCN-Bn-Desferrioxamine (SCN-Bn-DFO, Macrocyclics) by incubating ~ 700 µg purified F(ab)'2 fragments with 10x molar excess SCN-Bn-DFO dissolved in DMSO in 0.1 M NaHCO_3_ (1 hour, 37 **°**C, pH=9.0). The reaction mixture was purified on PD-10 desalting columns (GE Healthcare, USA) into PBS resulting in DFO-CD8a, DFO-CD4 and DFO-IgG2 precursor.

^89^Zr-oxalate (PerkinElmer, the Netherlands) was neutralized to pH ~7 with 1 M Na_2_CO_3_. Conjugated F(ab)'2 fragments (100 µg DFO-CD8a, DFO-CD4, and DFO-IgG2 precursor) were incubated with 150 MBq neutralized 89Zr-oxalate (1 hour, 37ºC, pH=7.0) followed by PD-10 purification into PBS. The radiochemical purity at end-of-synthesis was determined by radio-thin-layer chromatography (radio-TLC) using an eluent of 50 mM EDTA (pH 5.5) on silica gel 60 TLC plates, where the antibody construct remains at the baseline, while ^89^Zr^4+^ ions and [^89^Zr]-EDTA elute with the solvent front. All optimization experiments including optimization of dose, imaging time-point and *in vivo* depletion were conducted with ^89^Zr-DFO-CD8a.

### SDS-page

Full length antibody, purified F(ab)'2 and purified Fc fragments were diluted in NuPAGE LDS sample buffer (#NP0007, Invitrogen), heated at 70 °C for 10 min and loaded onto Bolt^TM^ 4-12% Bis-Tris gels (#NW04120BOX, Invitrogen). Electrophoresis was run on the Mini Gel Tank system (Life Technologies) at 200V constant voltage in NuPAGE MES SDS running buffer (#NP0002, Invitrogen). Gels were fixed and stained with Coomassie brilliant blue R-250 (#1610436, Bio-Rad).

### Mouse T cell isolation and immuno-reactivity

Murine CD4^+^ and CD8a^+^ cells were isolated from mouse spleen using the magnetic activating cell sorting (MACS) technique (CD4^+^ T cell isolation kit, #130-104-454; CD8a^+^ T cell isolation kit, #130-104-075, Miltenyi Biotec). Female BALB/c mice (7-8 weeks old) were sacrificed by cervical dislocation and the spleens removed aseptically. Spleens were mashed with a plunger through a moisturized 70 µm nylon mesh and rinsed in FACS buffer (1% BSA, 0.5 mM EDTA in PBS) until complete dissociation. Cells were pelleted and resuspended in red blood cell lysis buffer (#11814389001, Sigma Aldrich), washed in FACS buffer and incubated with biotin-antibody cocktail (4 °C, 5 min) followed by incubation with anti-biotin microbeads (4°C, 10 min). Magnetic separation was performed on a QuadroMACS^TM^ Manual Separator with a pre-rinsed LS column (#130-042-401, Miltenyi Biotec) based on negative selection methods.

The immuno-reactivity of anti-CD4 and anti-CD8a F(ab)'2 fragments following radiolabeling was assessed according to the Lindmo assay 27. Increasing concentrations of CD4^+^/CD8a^+^ cells (0.25x10^6^ - 4x10^7^ cells/mL) were incubated with 2 nM ^89^Zr-DFO-CD4 or ^89^Zr-DFO-CD8a for 3 hours at 4°C. Cells were centrifuged at 500g for 5 minutes and the supernatants and pellets counted in a gamma counter (Wizard^2^, PerkinElmer, Massachusetts, USA). Cell-associated radioactivity was calculated as the ratio of cell-bound radioactivity to the total amount of added radioactivity.

### Optimization of dose and imaging time-point

Titration of dose and longitudinal imaging was performed in CT26 tumor-bearing mice (150-200 mm^3^) and was only performed for ^89^Zr-DFO-CD8a. Mice were injected with ^89^Zr-DFO-CD8a (1.34 ± 0.1 MBq) without or with 5, 10, 30 or 100 µg unlabelled CD8a-F(ab)'2 intravenously (total volume ~ 200 µL in PBS, N=3/group). Small animal PET/CT imaging was performed 1, 4, 24 and 72 hours after injection on an Inveon Multimodality PET/CT scanner (Siemens, Germany). Mice were anesthetized with sevoflurane (3-4% in 80% N_2_, 20% O_2_) during PET/CT imaging. Static PET data were acquired in list mode with an acquisition time of 300, 300, 600 and 900 seconds for the 1, 4, 24 and 72 time-point, respectively. Images were reconstructed using a 3D maximum a posteriori algorithm with CT based attenuation correction. Image analysis (Inveon Software, Siemens) was performed by drawing CT based regions of interest (ROIs) over the tumor, whole heart, liver, kidney, muscle, inguinal lymph node (ILN), axillary lymph node (ALN) and cervical lymph node (CLN). ROIs over the spleen were drawn by PET based thresholding. The uptake of ^89^Zr-DFO-CD8a was quantified as % injected dose per gram tissue (%ID/g) assuming a soft tissue density of 1 g/cm^3^. Blood was withdrawn by cardiac puncture and mice were euthanized after the imaging session, organs resected, weighted and the radioactivity counted in a gamma counter.

### *In vivo* depletion

Mice bearing subcutaneous CT26 tumors (150-200 mm^3^) were treated for three consecutive days with intraperitoneal injections of either saline, 300 µg of CD8a^+^ depleting antibody clone 2.43, a full length rat-anti-mouse IgG2b (#BE0061, BioXcell), or 300 µg DFO-CD8a precursor (N=5/group). Single cell suspensions of blood, tumors and spleens were obtained according to the above-described method and stained for viability (#65-0865-18, ThermoFisher Scientific), CD45 (BV480 clone 30-F11, #566095), CD3 (PE, clone 145-2C11, #100308), CD4 (FITC, clone RM4-5, #553047) and CD8a (BV711, clone 53-6.7, #563046) (N=2/group). All antibodies were purchased from BD Biosciences. Analyses were conducted on a LSR-Fortessa flow cytometer (BD) and data analysed with FlowJo software (v10, TreeStar Inc., USA).

After end of therapy, saline and CD8a^+^ depleted mice (N=3/group) were injected with ^89^Zr-DFO-CD8a (1.97 ± 0.08 MBq) + 30 µg CD8a-F(ab)'2 intravenously. A group of naïve immunodeficient nude mice was included to determine distribution in antigen-negative mice. Mice were subjected to PET/CT imaging with 300 seconds static PET acquisition 24 hours after injection according to the above-described protocol. Mice were euthanized after the imaging session and underwent conventional *ex vivo* biodistribution analysis as described above.

### Isotype control imaging

Mice bearing subcutaneous CT26 tumors (150-200 mm^3^, N=3) were intravenously injected with ^89^Zr-DFO-IgG2b (2.2 - 2.6 MBq, 3.1 ± 0.1 µg) + 30 µg IgG2b-F(ab)'2 and subjected to PET/CT imaging with 300 seconds static PET acquisition 24 hours after injection according to the above-described protocol. Mice were euthanized after the imaging session and underwent conventional *ex vivo* biodistribution analysis as described above.

### External radiation therapy and autoradiography

Mice carrying CT26 subcutaneous tumors (150-200 mm^3^) were randomized into two groups (N=8/group) and subjected to 2 Gy external radiation therapy (XRT) for three consecutive days at a dose rate of 1 Gy/min (320 kV, 12.5 mA) using a small animal irradiator (XRAD-320, pXi, CT, USA). Mice were placed in the radiation chamber in a restrainer allowing total fixation of the leg and the body was covered by lead shielding so that only the tumor was exposed to radiation. On day 3 after the last radiation dose, mice were intravenously injected with ^89^Zr-DFO-CD8a (1.12 ± 0.11 MBq) + 30 µg CD8a-F(ab)'2 and subjected to PET/CT imaging with 300 seconds static PET acquisition 24 hours after injection according to the above-described protocol. Image analysis was performed by drawing CT based ROIs over the tumor and PET based thresholding over the spleen.

Following ^89^Zr-DFO-CD8a PET two mice from each group were subjected to perfusion fixation and tumors snap frozen in chilled isopentane in OCT medium for autoradiographic measurement. Tumors were cryosectioned at 10 µm and slides mounted on SuperFrost ULTRA PLUS slides. Intratumoral distribution of ^89^Zr-DFO-CD8a was determined by exposing tumor sections to phosphor imaging screens for approximately 16 hours. Phosphor screens were read on an Amersham Typhoon Imager (GE Healthcare, IL, USA). A parallel set of mice subjected to an identical treatment regimen (N=6/group) were euthanized, spleens and tumors isolated and processed into single cell suspensions, stained for viability (#65-0865-18, ThermoFisher Scientific), CD45 (BV480 clone 30-F11, #566095) and CD8a (BV711, clone 53-6.7, #563046) according to the above-described protocol. Data were collected on a LSR-Fortessa flow cytometer (BD) and analysed with FlowJo software (v10, TreeStar Inc., USA).

### Small animal PET/CT imaging and Sym021 treatment

Once animals representing each syngeneic mouse model (B16F10, P815, CT26, MC38, Renca, 4T1, Sa1N) reached an average tumor volume of 60-80 mm^3^ (8-10 days post inoculation) they were randomized into 3 treatment groups; ^89^Zr-DFO-CD4 (N=5), ^89^Zr-DFO-CD8a (N=5) and vehicle (N=5). ^89^Zr-DFO-CD4 (0.4-1.1 MBq, 2.1 ± 0.1 µg) and ^89^Zr-DFO-CD8a (0.3-0.7 MBq, 1.8 ± 0.3 µg) were injected intravenously and mice were subjected to PET/CT imaging with 300 seconds static PET acquisition 24 hours after injection according to the above-described protocol. Image analysis was performed by drawing CT based ROIs over the tumors and whole heart. Immunotherapy with Sym021 was initiated immediately after image acquisition. Sym021 was dosed intraperitoneally at 10 mg/kg 3x weekly for a total of 6 doses. Tumor growth was monitored 3x weekly for evaluation of therapeutic response and the tumor growth inhibition (TGI) was calculated relatively to the mean tumor volume of the control group to correct for inter-model tumor growth differences. TGI was expressed as % and calculated as ((average tumor volume(day 10)_control group_ - tumor volume(day 10)_treated mouse_)/ average tumor volume(day 10)_control group_) x 100. Mice were euthanized at the first tumor measurement above 1,500 mm^3^.

### Statistical analyses

Data are expressed as mean ± SEM. One-way ANOVA with *post hoc* test corrected for multiple comparisons (Tukey) was applied to test the effect of depletion and compare tumor volumes between treatment groups. Student's t-test was performed to test the effect of external radiation therapy and compare tumor-to-heart ratios between responders and non-responders. For presentation of mean tumor growth over time, carry-forward of tumor volumes was performed until the last day where >50% of the treatment group was alive to account for missing points. Two-way ANOVA with repeated measures was applied to compare the tumor volumes over time. Linear regression analyses were performed to test for correlations and the Log-rank test (Mantel-Cox) was applied for survival analyses.* P* values ≤ 0.05 were considered statistically significant. Statistical analyses were performed using GraphPad Prism 7.0c (GraphPad Software, CA, USA).

## Results

### Generation of radiolabelled F(ab)'2 fragments

**Figure 1:**
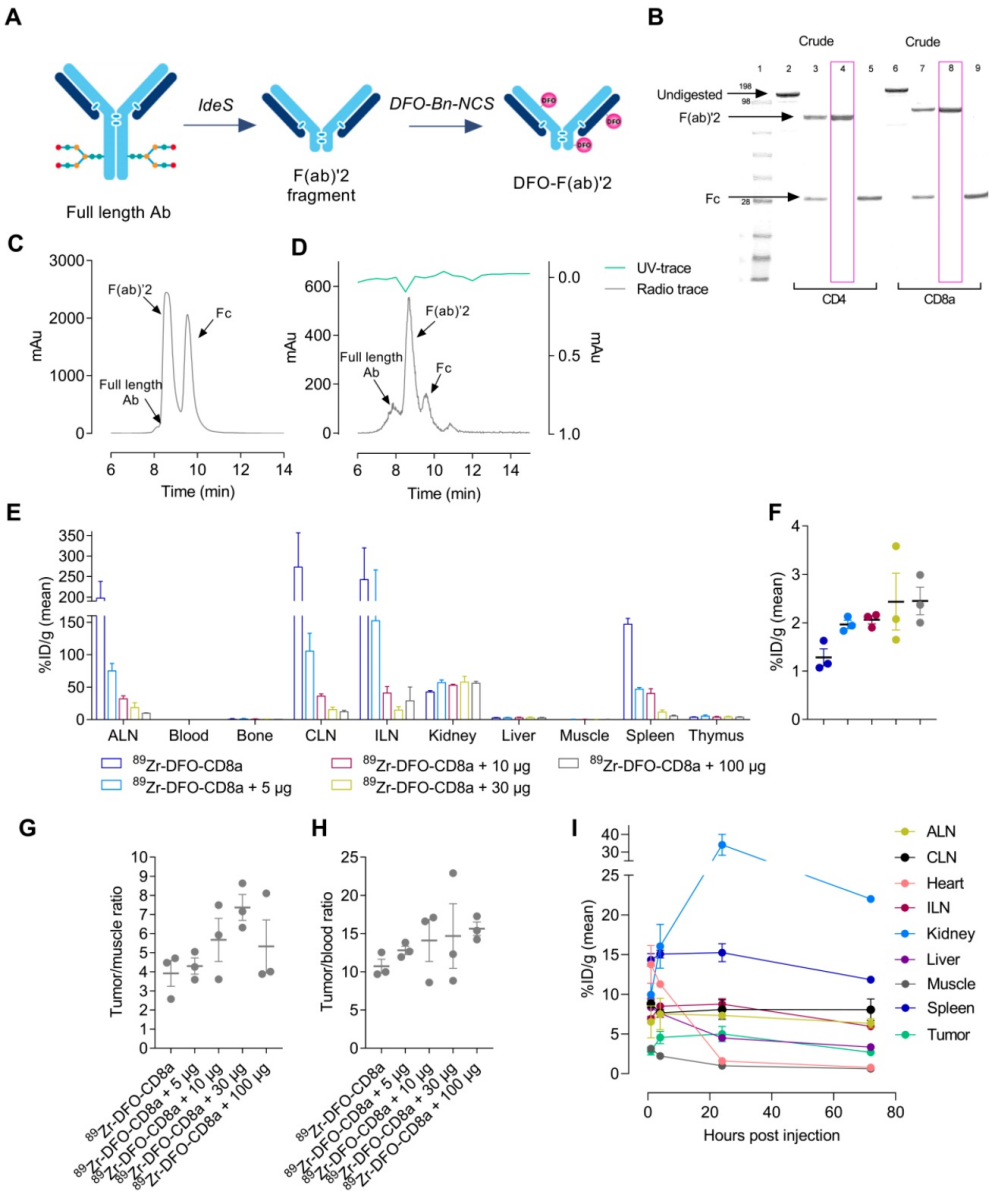
** Development and optimization of radiolabeled F(ab)'2 fragments for PET imaging**. (A) Schematic illustration of preparation of F(ab)'2 fragments from full length antibodies using FaBRICATOR (IdeS protease). (B) SDS-page of non-digested (lane 2+6), crude antibody mixture (lane 3+7) and purified F(ab)'2 (lane 4+8) and Fc fragments (lane 5+9) of anti-mouse CD4 and anti-mouse CD8a. (C) Representative HPLC chromatogram of crude anti-mouse CD8a antibody mixture used for preparative purification. (D) Representative HPLC chromatogram of ^89^Zr-DFO-CD8a after PD10 purification at end-of-synthesis. (E) *Ex vivo* biodistribution 72 hours post-injection of ^89^Zr-DFO-CD8a in major organs and (F) CT26 tumors with increasing doses of unlabeled CD8a-F(ab)'2 measured by gamma counting and expressed as %ID/g (N=3/dose). (G) Tumor-to-muscle and (H) tumor-to-blood ratio of uptake quantified by gamma counting 72 hours post-injection of ^89^Zr-DFO-CD8a (N=3/dose). (I) Image-derived biodistribution of dose-optimized mean ^89^Zr-DFO-CD8a uptake in major organs and tumor based on ROI analysis and expressed as mean %ID/g in CT26 tumor-bearing mice over the imaging time-course (N=3). Data are presented as mean ± SEM. Ab: antibody; ALN: axillary lymph node; CLN: cervical lymph node; ILN: inguinal lymph node; %ID/g: % injected dose per gram tissue.

Rat-anti-mouse-CD4, rat-anti-mouse-CD8a and rat-anti-mouse-IgG2b were successfully digested in the hinge region yielding F(ab)'2 and Fc fragments (**Figure 1**A). SDS-page analysis confirmed the digestion efficiency and revealed F(ab)'2 at 100 kDa (lane 4, 8) with no presence of Fc or full-length antibody in the final product (**Figure 1**B). F(ab)'2 fragments were conjugated to the SCN-Bn-DFO chelator and radiolabeled with ^89^Zr with a radiochemical yield of 11.9 ± 2.6, 17.5 ± 4.3 and 37 MBq for ^89^Zr-DFO-CD4, ^89^Zr-DFO-CD8a and ^89^Zr-DFO-IgG2b, respectively. All tracers were >99% pure as assessed by radio-TLC. Representative HPLC chromatograms of digested rat-anti-mouse-CD8a^+^ and ^89^Zr-DFO-CD8a are shown in **Figure 1**C-D, where a major UV peak for F(ab)'2 corresponding with the radioactive peak, and two minor peaks for full-length antibody and Fc were observed (**Figure 1**D). The immuno-reactivity of ^89^Zr-DFO-CD4 and ^89^Zr-DFO-CD8a towards CD4 and CD8a expressing splenocytes was estimated to 90.5 ± 0.6% and 86.7 ± 0.7%, respectively. Specifications of each tracer are summarized in **Table 1**.

### Optimization of imaging parameters

Radiolabeled F(ab)'2 fragments were initially evaluated in CT26 tumor-bearing mice to optimize imaging parameters for tumor visualization. ^89^Zr-DFO-CD8a was titrated with cold doses of CD8a-F(ab)'2 ranging from 0-100 µg where uptake was primarily seen in lymphoid tissue, i.e. spleen and lymph nodes. Retention in kidneys was also observed as expected for radiolabeled antibody fragments 28. Uptake in lymphoid tissue significantly decreased with increasing co-dose as evident from the representative PET images (**Figure S1**) and *ex vivo* biodistribution 72 hours post-injection (**Figure 1**E). Contrary, availability of ^89^Zr-DFO-CD8a for tumor accumulation was evident and uptake increased from 1.28 ± 0.17 to 2.45 ± 0.28 % injected dose per gram (%ID/g) with increasing dose of CD8a-F(ab)'2 (**Figure 1**F, **Table S1**). Importantly, the tumor-to-muscle (**Figure 1**G) and tumor-to-blood (**Figure 1**H) ratios also increased with increasing dose. However, a decrease in the tumor-to-muscle ratio of ^89^Zr-DFO-CD8a was observed at 100 µg (**Table S1**). Based on these results 30 µg was chosen as the optimal co-dose.

Temporal* in vivo* distribution of protein dose-optimized ^89^Zr-DFO-CD8a by longitudinal PET imaging at 1, 4, 24 and 72 hours post-injection is depicted in **Figure 1**I. High accumulation was seen in kidney, spleen and lymph nodes which increased over the imaging time-course. However, no further improvement in image contrast was seen beyond 24 hours (**Figure S2**). Tumor uptake was 2.88 ± 0.71, 4.55 ± 1.07, 5.0 ± 1.3 and 2.68 ± 0.56 % ID/g for the 1, 4, 24 and 72 hour time-points, respectively. Despite high kidney uptake, 24 hours post-injection was chosen as optimal for imaging due to measurable tumor growth resulting in dilution of tumor PET signal and limited gain in image contrast at 72 hours.

### Specificity of ^89^Zr-DFO-CD8a PET signal

**Figure 2:**
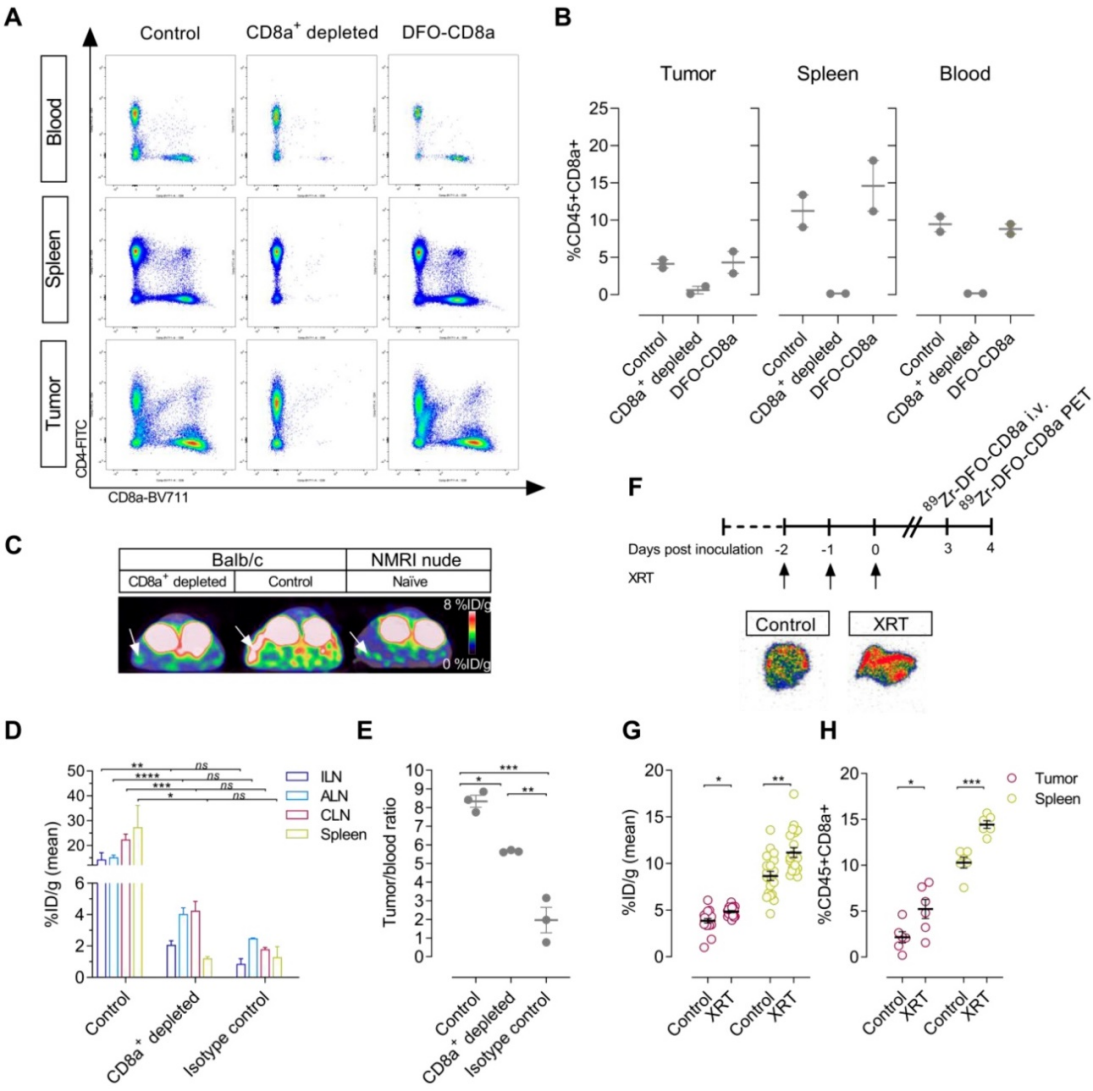
** Specificity of ^89^Zr-DFO-CD8a in depleted, antigen-negative and tumor-bearing mice.** (A) Representative dot plots of median fluorescent intensity of CD4-FITC (y-axis) and CD8a-BV711 (x-axis) measured by flow cytometric analysis of blood, spleen and tumors of control (N=2), CD8a^+^ depleted (N=2) and DFO-CD8a treated (N=2) CT26 tumor-bearing mice. (B) Depletion with CD8a 2.43 mAb reduced the percentage of CD45^+^CD8a^+^ cells in blood, spleen and tumor whereas DFO-CD8a precursor did not change CD45^+^CD8a^+^ populations (N=2/group). (C) Representative axial PET/CT images 24 hours post-injection of ^89^Zr-DFO-CD8a in control, CD8a^+^ depleted and NMRI nude (antigen-negative) mice. Arrows designate the spleen. (D) *Ex vivo* biodistribution of ^89^Zr-DFO-CD8a and ^89^Zr-DFO-IgG2b (isotype control) in lymphoid tissue. Mean ^89^Zr-DFO-CD8a uptake was significantly reduced in CD8a^+^ depleted mice (N=3/group). (E) The ^89^Zr-DFO-CD8a tumor-to-blood ratio was lowered in CD8a^+^ depleted mice compared to control mice (p=0.011) (N=3/group). The ^89^Zr-DFO-IgG2b (isotype control) tumor-to-blood ratio was significantly different than the ^89^Zr-DFO-CD8a tumor-to-blood ratio in control (p=0.0001) and CD8a^+^ depleted mice (p=0.0024). (F) Autoradiography of tumors showed increased ^89^Zr-DFO-CD8a uptake in tumors subjected to fractionated external radiation therapy (XRT, 3x2Gy). (G) XRT (3x2Gy) increased the mean ^89^Zr-DFO-CD8a uptake in tumors (p=0.0006) and spleens (p=0.0022) of CT26 tumor-bearing mice (N=16/group) that was confirmed by (H) flow cytometric analysis of CD45^+^CD8a^+^ cells (N=6/group). Data are presented as mean ± SEM and the significance levels are indicated by asterisks (*). *=p<0.05, **=p<0.01, ***=p<0.001, ****=p<0.0001, ns=no significance. ALN: axillary lymph node; CLN: cervical lymph node; ILN: inguinal lymph node; XRT: external radiation therapy; %ID/g: % injected dose per gram tissue.

To evaluate the specificity of ^89^Zr-DFO-CD8a towards CD8a, CT26 tumor-bearing mice were treated with CD8a^+^ depleting antibody. Furthermore, to ensure that ^89^Zr-DFO-CD8a does not perturb CD8a^+^ populations *in vivo*, a group of mice received equally high doses of DFO-CD8a precursor. Flow cytometric analyses showed a >95% reduction in CD45^+^CD8a^+^ cells in blood and spleens and a ~85% reduction in the tumors of mice treated with CD8a^+^ depleting mAb (**Figure 2**A-B). Importantly, high dose DFO-CD8a precursor did not alter CD45^+^CD8a^+^ populations in any of the analysed tissues. ^89^Zr-DFO-CD8a PET was able to detect the loss of CD45^+^CD8a^+^ cells in the spleen as visualized by the representative PET/CT images (**Figure 2**C). When injected into antigen-negative mice lacking mature T and B lymphocytes, ^89^Zr-DFO-CD8a uptake in spleen was similar to that of CD8a^+^ depleted mice confirming the specificity of ^89^Zr-DFO-CD8a (**Figure 2**C). *Ex vivo* biodistribution confirmed a significant decrease in ^89^Zr-DFO-CD8a uptake in spleen (*p<0.05*) and lymph nodes (*p<0.01*) of CD8a^+^ depleted mice (**Figure 2**D). Uptake of ^89^Zr-labeled isotype control antibody (^89^Zr-DFO-IgG2b) in lymphoid tissue was low (~1-3 %ID/g) and was similar to the ^89^Zr-DFO-CD8a uptake in CD8a^+^ depleted mice (*p=0.55*). To compare the accumulation of ^89^Zr-DFO-CD8a and ^89^Zr-DFO-IgG2b in tumors, the tumor uptake was normalized to the blood pool in order to account for an observed difference in circulation time between the probes. The tumor-to-blood ratio of ^89^Zr-DFO-CD8a was significantly lower in CD8a^+^ depleted mice compared to control mice (5.6 ± 0.03 vs. 8.3 ± 0.32, *p=0.011*, **Figure 2**E). In addition, the tumor-to-blood ratio of ^89^Zr-DFO-IgG2b was 1.9 ± 0.68 and significantly lower than that of control (*p=0.0001*) and CD8a^+^ depleted (*p=0.0024*) mice.

Fractionated XRT was applied to CT26 tumor-bearing mice to investigate whether treatment-induced changes in CD8^+^ populations 29 could be detected with the probes (**Figure 2**F). XRT induced lymphocyte infiltration in tumors and spleens of CT26 tumor-bearing mice that was detected by ^89^Zr-DFO-CD8a PET/CT (**Figure 2**G, **Figure S3**). Mean tumor uptake increased from 3.8 ± 0.2 to 4.9 ± 0.1 %ID/g (*p=0.0006*) and mean spleen uptake increased from 8.7 ± 0.5 to 11.2 ± 0.5 %ID/g (*p=0.0022*). *Ex vivo* autoradiographic measurement of ^89^Zr-DFO-CD8a visualized this distribution pattern in tumors between treated and non-treated groups (**Figure 2**F). Further, flow cytometric analysis confirmed the increased numbers of CD45^+^CD8a^+^ cells in tumors (*p=0.0287*) and spleens (*p=0.0002*) of irradiated mice (**Figure 2**H).

### Characterization of syngeneic mouse tumor models

**Figure 3:**
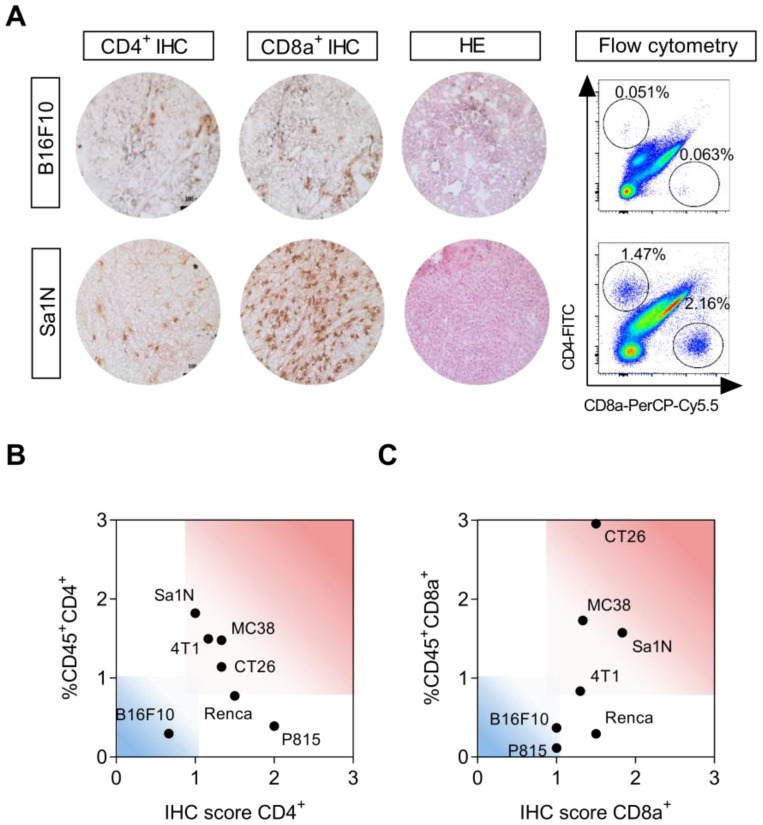
** Characterization of syngeneic mouse models by CD4^+^ and CD8a^+^ subsets.** (A) Representative sections from immunohistochemical (IHC) staining of CD4^+^ and CD8a^+^, and hematoxylin and eosin (HE) staining in a tumor model with low (B16F10) and high (Sa1N) intensity staining (left panel). Representative dot plots of median fluorescent intensity of CD4-FITC (y-axis) and CD8a-PerCP-Cy5.5 (x-axis) of flow cytometric analysis of CD4^+^ and CD8a^+^ cells in a tumor model with low (B16F10) and high (Sa1N) intensity staining (right panel). (B) Agreement between flow cytometric and IHC analysis of CD4^+^ and (C) CD8a^+^ subsets revealed clustering of tumor types into hot (red) and cold (blue) areas. N=6/model for flow cytometry, N=3/model for IHC. HE: hematoxylin and eosin; IHC: immunohistochemistry.

Immune status of a panel of seven subcutaneous syngeneic mouse tumor models was analysed by immunohistochemistry and flow cytometry with respect to their capacity to attract TILs, i.e. CD4^+^ and CD8a^+^ cells. Representative immunohistochemical images and flow cytometric plots are shown in **Figure 3**A, representing a model with low (B16F10) and high (Sa1N) numbers of CD4^+^ and CD8a^+^ cells.

Varying degree of lymphocyte infiltration was observed and tumor types were stratified into immunologically “hot” or “cold” tumors based on their numbers of CD4^+^ and CD8a^+^ cells 12,30-32. Sa1N, MC38, CT26, and 4T1 presented with the highest number of infiltrating CD4^+^ and CD8a^+^ T cells and were defined as “hot” tumors (**Figure 3**B-C, read area). The lowest numbers of CD4^+^ and CD8a^+^ cells were found in B16F10 (**Figure 3**B-C, blue area), defined as a “cold” tumor, and intermediate T cell numbers were found in Renca and P815, although high CD4^+^ numbers were found in both models based on IHC score only. Quantitative analysis of all models is illustrated in **Figure S4** and summarized in **Table 2**.

### CD4^+^ and CD8a^+^ PET imaging in syngeneic mouse models

**Figure 4:**
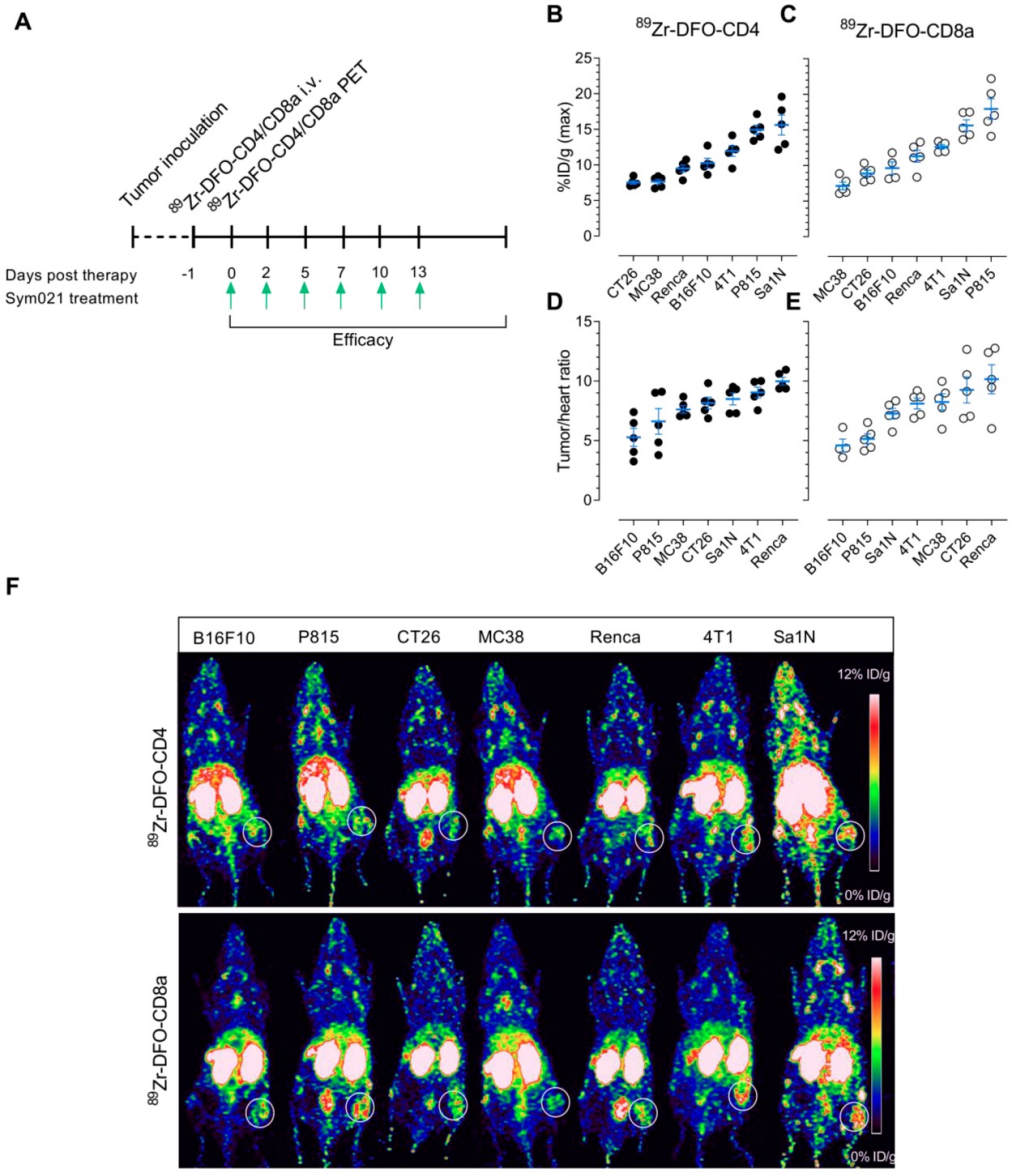
** Experimental design and PET imaging of ^89^Zr-DFO-CD4 and ^89^Zr-DFO-CD8a in a panel of syngeneic mouse models.** (A) Overview of the timing of tumor inoculation, ^89^Zr-DFO-CD4 and ^89^Zr-DFO-CD8a injections, PET imaging and therapy dosing. Sym021 (10 mg/kg) was dosed 6 times over two weeks and efficacy monitored until humane endpoints were reached. (B) Maximum ^89^Zr-DFO-CD4 and (C) ^89^Zr-DFO-CD8a uptake in syngeneic mouse models quantified from PET ROI analysis of tumors and expressed as %ID/g 24 hours post-injection of tracer ranked from low (left) to high (right). Tumor-to-heart ratios of the maximum (D) ^89^Zr-DFO-CD4 and (E) ^89^Zr-DFO-CD8a uptake quantified from PET ROI analysis and expressed as %ID/g 24 hours post-injection of tracer ranked from low (left) to high (right). (F) Representative coronal maximum intensity projection PET images of ^89^Zr-DFO-CD4 (top panel) and ^89^Zr-DFO-CD8a (bottom panel) for each model. The PET acquisition time was 300 seconds. White circles designate the tumor. Data are presented as mean ± SEM. %ID/g: % injected dose per gram tissue.

Baseline PET imaging of CD4^+^ and CD8a^+^ was conducted in the same panel of syngeneic mouse models prior to treatment with Sym021 to evaluate the predictive value of TIL imaging for treatment response across various tumor types (**Figure 4**A). Quantitative ROI analysis of ^89^Zr-DFO-CD4 maximum tumor uptake is depicted in **Figure 4**B ranked from high to low and revealed uptake levels of 15.6 ± 1.4 (Sa1N), 14.9 ± 0.65 (P815), 11.9 ± 0.74 (4T1), 10.2 ± 0.67 (B16F10), 9.5 ± 0.5 (Renca), 7.65 ± 0.35 (MC38) and 7.53 ± 0.25 (CT26) %ID/g. Likewise, ^89^Zr-DFO-CD8a uptake is illustrated in **Figure 4**C and was 17.9 ± 1.4 (P815), 15.6 ± 0.8 (Sa1N), 12.6 ± 0.3 (4T1), 11.3 ± 0.84 (Renca), 9.6 ± 0.9 (B16F10), 8.9 ± 0.5 (CT26) and 7.1 ± 0.5 (MC38) %ID/g. Tumor-to-heart ratios (tumor_max_/heart_mean_) were calculated to correct for activity remaining in the blood and were 9.99 ± 0.3 (Renca), 9.05 ± 0.4 (4T1), 8.49 ± 0.44 (Sa1N), 8.15 ± 0.44 (CT26), 7.62 ± 0.28 (MC38), 6.61 ± 0.96 (P815) and 5.28 ± 0.68 (B16F10) for ^89^Zr-DFO-CD4 (**Figure 4**D). Similarly, the ^89^Zr-DFO-CD8a tumor-to-heart ratios are illustrated in **Figure 4**E and were 10.15 ± 1.09 (Renca), 9.25 ± 0.98 (CT26), 8.25 ± 0.6 (MC38), 8.11 ± 0.41 (4T1), 7.26 ± 0.4 (Sa1N), 5.15 ± 0.38 (P815) and 4.6 ± 0.67 (B16F10). Representative maximum intensity projection PET images for each model are shown in **Figure 4**F, where ^89^Zr-DFO-CD4 and ^89^Zr-DFO-CD8a clearly visualized the tumors 24 hours post-injection. Representative axial PET/CT images of the tumor and heart for each model are shown in **Figure S5**. Mean tumor uptake and tumor-to-heart (tumor_mean_/heart_mean_) ratios are depicted in **Figure S6**A-B. Overall, the mean and maximum tumor-to-heart ratios of ^89^Zr-DFO-CD4 and ^89^Zr-DFO-CD8a were in agreement with flow cytometric analysis of CD4^+^ (**Figure S6**C-D, top panel) and CD8a^+^ (**Figure S6**C-D, bottom panel) numbers across syngeneic mouse tumor models.

### Tumor growth response to Sym021

**Figure 5:**
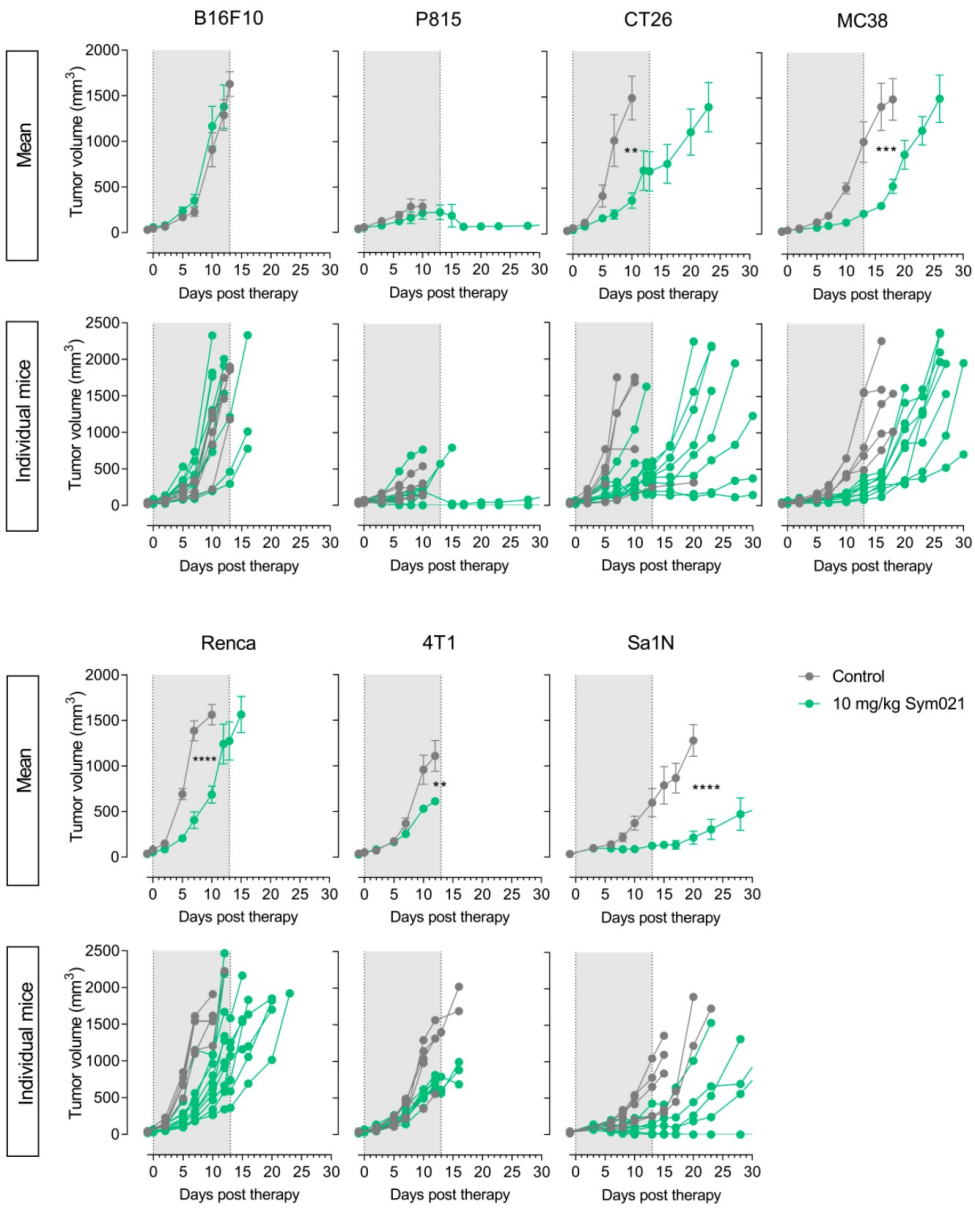
** Efficacy of immunotherapy with Sym021 in syngeneic mouse models.** Therapy with Sym021 (green) at 10 mg/kg over two weeks effectively inhibited tumor growth in CT26 (p=0.0029), MC38 (p=0.0002), Renca (p<0.0001), 4T1 (p=0.0011) and Sa1N (p<0.0001) compared to vehicle-treated animals (grey). No effect of Sym021 was observed in B16F10 (p=0.49) and P815 (p=0.1721) tumor models. The grey area indicates the treatment period. Mice were euthanized after first tumor measurement above 1,500 mm^2^ except in the case of the P815 and 4T1 model, where humane endpoints were reached prematurely. Mean tumor volumes of treatment groups are presented with carry-forward of tumor volumes from euthanized mice until the last day where >50% of the group was alive. N=5 for vehicle-treated animals, N=10 for Sym021 treated animals. Data are presented as mean ± SEM and the significance levels are indicated by asterisks (*). * = p<0.05, ** = p<0.01, *** = p<0.001, **** = p<0.0001.

Following PET imaging, mice were subjected to immunotherapy with Sym021. All groups, within each model and across models, had equal mean tumor volumes at day -1 relative to start of therapy (**Figure S7**). Tumor growth curves for Sym021 treated mice (N=10/group) are shown in **Figure 5** for each model and were compared to corresponding vehicle-treated animals (N=5/group) that were not subjected to CD4^+^ or CD8a^+^ PET imaging. Sym021 effectively inhibited tumor growth in CT26 (*p=0.0029*), MC38 (*p=0.0002*), Renca (*p<0.0001*), 4T1 (*p=0.0011*) and Sa1N (*p<0.0001*) at 10 mg/kg. No overall effect of Sym021 was found in B16F10 (*p=0.49*) and P815 (*p=0.1721*) tumor models. Mice were terminated when reaching humane endpoints or at study end at day 65, where no signs of tumor regrowth was apparent. Due to issues with wound formation and/or necrosis in certain models humane endpoints were reached prematurely for P815 (~500 mm^3^) and 4T1 (~1000 mm^3^).

It is noteworthy, that inter-model efficacy seemed to be overall associated with the immune status, since models with the highest number of CD4^+^ and CD8a^+^ cells according to combined IHC, flow cytometry and PET tumor-to-heart ratios (Sa1N, 4T1, CT26 and MC38), demonstrated significantly reduced tumor growth in response to Sym021 (**Figure 3**B-C). In contrast, models with the lowest numbers of CD4^+^ and CD8a^+^ cells (B16F10 and P815) exhibited no response to Sym021.

### Sym021 efficacy correlates with PET imaging of tumor-infiltrating lymphocytes

**Figure 6:**
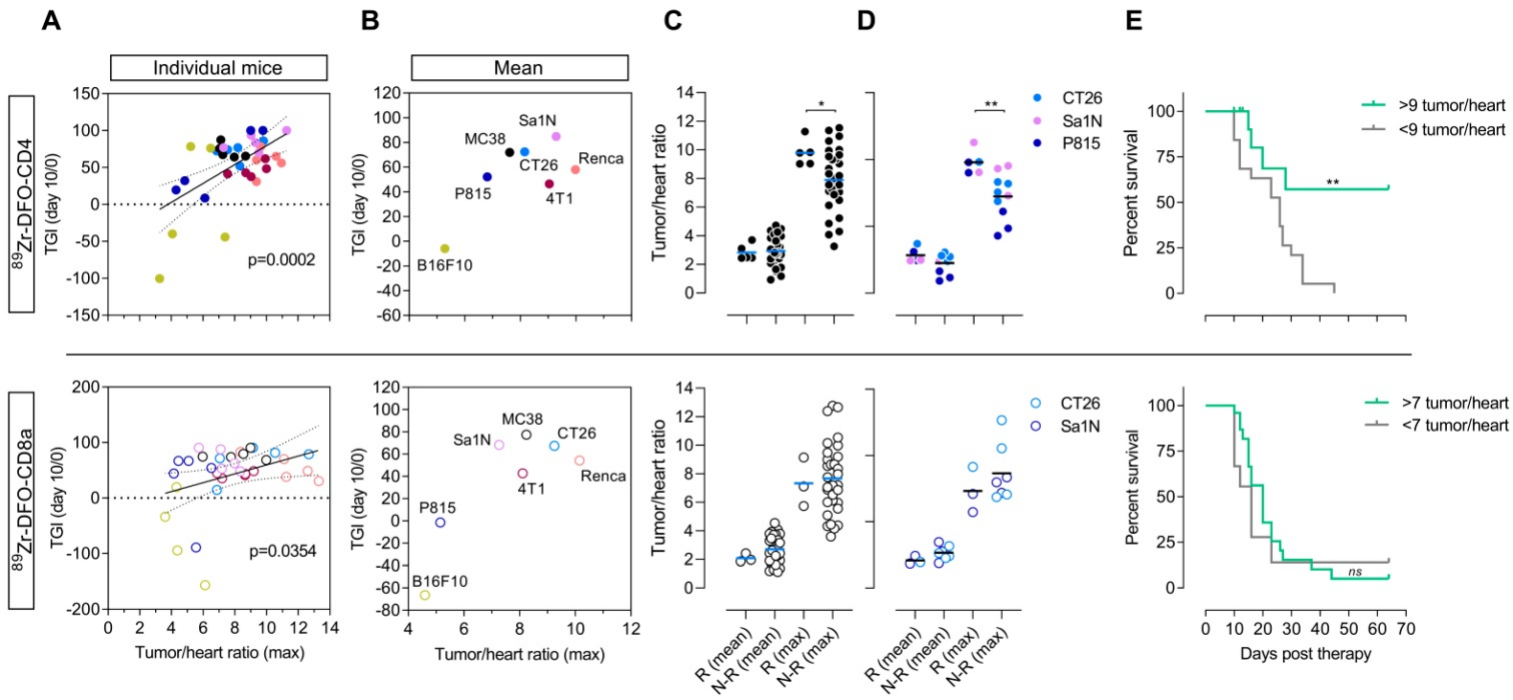
**^89^Zr-DFO-CD4 PET predicts Sym021 treatment outcome in syngeneic mouse models.** (A) The tumor growth inhibition (TGI) from day 0 til day 10 in Sym021 treated mice (10 mg/kg) relative to the growth of the control group expressed as % correlated with the maximum tumor-to-heart ratio of ^89^Zr-DFO-CD4 (p=0.0002, top panel) and ^89^Zr-DFO-CD8a (p=0.0354, bottom panel) in individual mice across all models (N=35/tracer). (B) Average TGI until day 10 relative to start of therapy with 10 mg/kg Sym021 (day 0) expressed as % for each model plotted against the average maximum tumor-to-heart ratio of ^89^Zr-DFO-CD4 (top panel) and ^89^Zr-DFO-CD8a (bottom panel) for each model (N=35/tracer, N=5/model). (C) Mean and maximum tumor-to-heart ratio of ^89^Zr-DFO-CD4 (top panel) and ^89^Zr-DFO-CD8a (bottom panel) in responders (Rs) and non-responders (N-Rs) across all models (N=35/tracer). Responders (N=5) had higher maximum ^89^Zr-DFO-CD4 tumor-to-heart ratio than all non-responders (N=30) (p=0.0306, top panel). (D) Mean and maximum tumor-to-heart ratio of ^89^Zr-DFO-CD4 (top panel) and ^89^Zr-DFO-CD8a (bottom panel) in responders and non-responders of the models where responders were found. Responders (N=5) had higher maximum ^89^Zr-DFO-CD4 tumor uptake than the non-responders of the models were responders were found (N=10) (p=0.0097, top panel). (E) Stratification of mice across all models based on their maximum tumor-to-heart ratio of ^89^Zr-DFO-CD4 (top panel) and ^89^Zr-DFO-CD8a (bottom panel) resulted in improved overall survival of mice with a maximum ^89^Zr-DFO-CD4 tumor-to-heart ratio >9 (N=5) compared to the <9 group (N=30) (p=0.0018). All tumor and heart uptake values of ^89^Zr-DFO-CD4 and ^89^Zr-DFO-CD8a were derived from PET ROI analysis and expressed as %ID/g. Data are presented as mean ± SEM and the significance levels are indicated by asterisks (*). * = p<0.05, ** = p<0.01, *** = p<0.001, **** = p<0.0001, ns = no significance. N-Rs: non-responders; ROI: region of interest; Rs: responders; %ID/g: % injected dose per gram tissue.

To investigate the predictive value of ^89^Zr-DFO-CD4 and ^89^Zr-DFO-CD8a the TGI from day 0 to day 10 in Sym021 treated mice relative to the growth of the control group was compared to the maximum baseline tumor-to-heart ratio of ^89^Zr-DFO-CD4 and ^89^Zr-DFO-CD8a. Day 10 was the latest day post-therapy initiation with sufficient mice in the control group across models. The relative tumor growth against PET tumor-to-heart ratio was plotted for individual mice across all tumor models (**Figure 6**A) as well as for the mean of each tumor model (**Figure 6**B). Color coding of the different models clearly demonstrated the heterogeneity within each model and across models. The maximum ^89^Zr-DFO-CD4 (*p=0.0002*) and ^89^Zr-DFO-CD8a (*p=0.0354*) tumor-to-heart ratio were found to correlate with the relative TGI at day 10 post therapy initiation, although the correlation appeared stronger for ^89^Zr-DFO-CD4. There was no association between the mean ^89^Zr-DFO-CD4 (*p=0.0817*) or ^89^Zr-DFO-CD8a (*p=0.0651*) tumor-to-heart ratio and the relative TGI at day 10 post therapy initiation (**Figure S8**).

### ^89^Zr-DFO-CD4 predicts treatment outcome

As evident from the tumor growth curves in **Figure 5**, three of the Sym021 responding tumor models showed pronounced effect on tumor growth with 1-2 responders in each model that did not regrow during the entire study period of 65 days. Responders among mice subjected to pre-therapy ^89^Zr-DFO-CD4 PET were found in the CT26 (N=1), Sa1N (N=2) and P815 (N=2) models. Responders among mice subjected to pre-therapy ^89^Zr-DFO-CD8a PET included CT26 (N=1) and Sa1N (N=2). When comparing the mean and maximum ^89^Zr-DFO-CD4 tumor-to-heart ratios of responders (N=5) and non-responders among all mice (N=30), irrespective of tumor model, a significantly increased maximum ^89^Zr-DFO-CD4 tumor-to-heart ratio was found in the responders (*p=0.0306*) (**Figure 6**C, top panel). This difference was more prominent when comparing the intra-model responders (N=5) with non-responders (N=10, *p=0.0097*) (**Figure 6**D, top panel). No difference in the ^89^Zr-DFO-CD4 mean tumor-to-heart ratio was found when comparing responders with non-responders among all mice (*p=0.8298*) or intra-model mice (*p=0.1529*). Further, no difference between the maximum ^89^Zr-DFO-CD8a tumor-to-heart ratio in responding mice (N=3) was found when comparing to all non-responding mice *(*N=32*, p=0.8248*) nor intra-model mice (N=12, *p=0.9352*) (**Figure 6**C-D, bottom panel).

All mice in the baseline ^89^Zr-DFO-CD4 responding group presented with a maximum tumor-to-heart ratio >9 (average 9.79 ± 0.37), whereas the non-responding group presented with a maximum ^89^Zr-DFO-CD4 tumor-to-heart ratio of 7.73 ± 0.35. We therefore speculated whether the maximum ^89^Zr-DFO-CD4 tumor-to-heart ratio could be used to stratify mice into subgroups that respond to Sym021 and predict overall survival. Based on the maximum ^89^Zr-DFO-CD4 tumor-to-heart ratio in the Sym021 responding group, 9 was chosen as cut-off value in a retrospective analysis. Overall survival was improved in mice with a tumor-to-heart ratio >9 of ^89^Zr-DFO-CD4 (*p=0.0018*), with a median survival of 51 days compared to 23 days in the <9 group (**Figure 6**E, top panel). A similar analysis was performed for ^89^Zr-DFO-CD8a, where the tumor-to-heart ratio of the responding group was 7.32 ± 0.63. Overall survival was not improved in mice with a tumor-to-heart ratio >7 of ^89^Zr-DFO-CD8a (*p=0.5037*), with a median survival of 20 days compared to 16 days in the <7 group (**Figure 6**E, bottom panel).

## Discussion

Immunotherapy has recently emerged as a particularly effective strategy for treating several types of cancer. Sym021 is a newly developed, humanized antibody targeting the PD-L1/PD-1 interaction between tumor cells and cells of the immune system, boosting the reactivity of primarily PD-1 expressing T lymphocytes towards tumor cells 33. Alongside the rapid development of immune-targeting agents the necessity of developing methods for reliably identifying patients that would respond to therapy becomes apparent. Companion diagnostic methods should preferably be non-invasive and sensitive to the dynamic changes in immune populations to ease the translation into clinical use. In the present study, we developed specific tracers for non-invasive PET imaging of CD4^+^ and CD8a^+^ cells to enable quantification of these lymphocytes on a whole-body level in mice. We applied the developed PET procedures to seven different syngeneic mouse tumor models and demonstrate that ^89^Zr-DFO-CD4 can be a predictor of tumor growth response and overall survival to Sym021.

After successful radiolabeling the tracers retained *in vitro* binding to target with immuno-reactivities > 85%. *In vivo* studies with ^89^Zr-DFO-CD8a showed effective targeting to CD8a^+^ tissue, i.e. lymph nodes and spleen in antigen-positive mice. The accumulation of tracer in lymphoid tissue was extremely high as expected for antigen-dense tissues. This phenomenon, commonly referred to as the antigen-sink, typically results in faster clearance at low antibody doses 34. We therefore titrated the total protein dose to saturate endogenous target and increase the availability of the tracer for tumor accumulation. Co-dosing with CD8a-F(ab)'2 increased the tumor uptake ~2-3 fold, which is quite substantial considering the relatively low target-abundance as opposed to tumor surface markers. The co-dosing strategy in the development of antibody-based PET tracers is increasingly debated. To visualize a target that is more abundant in non-tumor tissues is not a common approach in the utilization of PET within oncology. Therefore, a dose optimization can be essential to obtain sufficient imaging signal, however, with the risk of also perturbing the binding of tracers to intratumoral T cells. Importantly, the 30 µg co-dose did not block tumor uptake as illustrated by the tumor-to-background ratios in this study. In addition, the splenic uptake was not blocked completely (12.17 ± 2.15 %ID/g, 30 µg) when comparing to non-blocked mice (147.9 ± 8.1 %ID/g*)*. Thus, a good balance between blocking the antigen sink without displacing tumor uptake was achieved.

A similar uptake in spleen and lymph nodes was previously shown by Tavaré *et al.,* who developed CD8a-targeting antibody-based PET probes of similar size, such as the ^64^Cu-NOTA-Mbs (~80 kD) 35. Compared to ^64^Cu-NOTA-Mbs, 89Zr-DFO-CD8a exhibited higher kidney accumulation (~4-6 vs. 16 ± 3.9 %ID/g, 4 hours p.i.). This can be explained by the higher protein dose injected (4-12 µg ^64^Cu-NOTA-Mbs vs. 30 µg ^89^Zr-DFO-CD8a), which can lead to a higher aggregation state since high antibody concentrations can promote protein association and thus kidney trapping 36-38. Furthermore the longer circulation time of ^89^Zr-DFO-CD8a compared to ^64^Cu-NOTA-Mbs (11.3 ± 0.09 vs. ~1 %ID/g, 4 hours p.i.) probably contributes to the kidney uptake due to the highly perfused nature of this organ. Lastly, the low pH environments that may exist in the kidneys and Kupffer cell lysosomes have been suggested to contribute to transchelation of ^89^Zr at these sites 39.

The target-specificity was further examined by depletion studies, where the lowered number of CD8a^+^ cells in tumors and spleens of CD8a^+^ depleted mice as confirmed by flow cytometry, was detected by ^89^Zr-DFO-CD8a PET. However, CD8a^+^ depleted mice accumulated some degree of tracer in tumors despite normalizing to the blood pool. Reasons for this could be multi-fold. PET imaging was not conducted in the same mice as used for flow cytometry and even though all mice were treated equally with CD8a^+^ depleting antibody depletion might have been insufficient in some mice. Another causative factor could be the enhanced permeability and retention (EPR) effect contributing to non-specific uptake of larger proteins such as antibodies and fragments hereof in tumors 40. The tumor-to-blood ratio of ^89^Zr-DFO-IgG2b was ~ 2 and represents the non-specific uptake of ^89^Zr-DFO-CD8a. Adding to this, an inflamed tumor microenvironment created by high therapeutic doses of immune-perturbing antibody could also contribute to a further increased permeability and retention of ^89^Zr-DFO-CD8a in tumors 41,42.

CD8a^+^ is mainly expressed on effector T cells but can also be found on regulatory T cells, natural killer cells and dendritic cells 43,44, all of which can be found at an early disease state in the tumor microenvironment 45,46. This is also in line with previous studies suggesting that agents targeting immune populations might have pleiotropic effects on T cell dynamics as well as inducing immune cell trafficking into tumors 47. It is crucial to the development of new imaging agents that they are biologically inert, especially when targeting immune cells. Importantly, we confirmed that our F(ab)'2 based agents lacking the Fc domain do not deplete CD8a^+^ cells *in vivo* and thus do not display any Fc-dependent biological activity, as expected.

The ability of ^89^Zr-DFO-CD8a to detect changes in response to radiation therapy was not limited to tumoral CD8a^+^ cell infiltration, but whole-body effects such as increased splenic infiltration were also detected, which was confirmed by flow cytometric analysis. These results underline the additive value of whole-body visualization with PET imaging, when monitoring dynamic populations such as trafficking T lymphocytes.

Unlike spontaneous-derived tumors in humans, syngeneic tumors are often engrafted subcutaneously in a microenvironment that is distinct from the site of origin and progress more rapidly than most spontaneous human tumors. Despite these differences from human cancer, each syngeneic tumor model is likely to be more or less immunogenic and have a unique immunophenotype that may reflect tumor profiles in humans. Indeed, studies of the immune infiltrate in subcutaneous versus orthotopic tumors in mice have shown differences in numbers, subtypes and the distribution of immune cell populations, suggesting that the efficacy of tumor-immune modulating agents might be different between inoculation sites 48. The level of CD4^+^ and CD8^+^ infiltration however, has been shown to be similar between subcutaneous and orthotopic colon or renal cancer models 49,50. Nevertheless, orthotopic tumors are generally considered to be more immuno-suppressive in nature and less sensitive to immunotherapy than their subcutaneous counterparts 48. Here we assessed the T cell infiltrate in multiple syngeneic mouse models representing various types of solid cancers of colon, breast, kidney and skin - tumor types among which PD-1/PD-L1 blockade has shown clinical success 51-53. Syngeneic tumor models were initially characterized by common *ex vivo* methods, IHC and flow cytometry of tumors, and classified into “hot” and “cold” tumors. The hot and highly immunogenic tumor models (Sa1N, CT26, MC38 and 4T1) were responsive to Sym021 treatment. The cold and poorly immunogenic tumor models B16F10 and P815 did not respond to Sym021 treatment. This response to immune checkpoint therapy as well as relative CD4^+^ and CD8a^+^ tumor numbers in syngeneic tumor models were similar to reported elsewhere 54-57.

When merely looking at the PET tumor uptake of ^89^Zr-DFO-CD4 and ^89^Zr-DFO-CD8a across syngeneic mouse models, imaging of CD4^+^ and CD8a^+^ cells did not completely match the assessment of immune status by IHC and flow cytometry in this study with discrepancies mainly found among the immunogenic CT26 and MC38 models (low uptake) and poorly immunogenic P815 (high uptake) model. However, when correcting for background levels of tracer, we found a good agreement between the mean and maximum tumor-to-heart ratios and flow cytometric analysis of CD4^+^ and CD8a^+^, which makes sense from a physiological perspective when assessing different tumor types. Several factors besides target abundance are known to influence tumor accumulation of compounds and larger molecules, such as micro-vessel density, vascular permeability, stromal content, intra-tumoral pressure and diffusion. Different tumor types display a great deal of heterogeneity. Moreover, a large degree of heterogeneity is also found within tumors of the same type. Indeed, tumor growth responses to anti-PD-1 antibody Sym021 therapy were rather heterogeneous across models and within models consistent with the variable therapeutic responses to monotherapy with PD-1 inhibitors seen among patients 58. For these reasons, all concurrent analyses of the utility of ^89^Zr-DFO-CD4/^89^Zr-DFO-CD8a PET as predictors of immunotherapeutic response were based on the background corrected tumor-to-heart values.

We utilize here a diverse panel of syngeneic tumors to represent the heterogeneity found within patient tumors with respect to differences in vascularization, contributions to the EPR effect and response to PD-1 checkpoint inhibition, and found that maximum tumor-to-heart values of ^89^Zr-DFO-CD8a and to a larger degree ^89^Zr-DFO-CD4 were indicators of response to therapy. Interestingly, no association between the mean tumor-to-heart ratios of ^89^Zr-DFO-CD4 or ^89^Zr-DFO-CD8a and tumor growth response to therapy was found. Maximum values of tumor imaging markers are the preferred choice clinically and have become the de facto standard 59. Additionally, they largely relate more strongly to prognosis as they reflect the most aggressive phenotype of the tumor 60,61. Moreover, in the context of immune cell markers maximum values might be even more applicable as immune cells are more dynamic, migrate and cluster at inflamed sites. In addition, it was demonstrated that when individual tumors were divided into groups with a maximum tumor-to-heart ratio above or below 9, ^89^Zr-DFO-CD4 stratified the population into responders and non-responders to Sym021 therapy. Yet, the fraction of responders was limited to 5 out of 35 treated mice distributed among 3 models and the predictivity of ^89^Zr-DFO-CD4 PET thus might be limited to certain tumor types.

To the best of our knowledge this is the first report on the predictive value of CD4^+^ PET imaging in oncology mouse models. A ^89^Zr-labeled CD4^+^ cDb has been evaluated in naïve 23 and colitis 62 mice as well as a model of hematopoietic stem cell transplantation 63. Probes targeting CTLA-4 20,64, which is generally expressed on activated T cells, and CD3^+^ 22, a global T cell marker, have shown success in discriminating responders from non-responders after immunotherapy was initiated. In contrast, attention in other previous studies has focused on the cytotoxic CD8^+^ T cells. In these studies it was also indicated that immuno-PET strategies were successful with correlating post-therapy CD8^+^ PET imaging and response to immunotherapy in a few animal models 24,25,65. Also, the CD8^+^-targeting minibody ^89^Zr-IAB22M2C is currently being tested clinically in patients undergoing immunotherapy 66. The radiotracers developed in our study are targeting murine CD4^+^ and CD8a^+^ cells. Accordingly, our probes can be used in preclinical studies using syngeneic tumor models enabling investigation of immunotherapy drugs in a host with a fully functional immune system. Currently, the majority of preclinical work within immuno-oncology development is indeed undertaken in such syngeneic tumor models and therefore we believe our probes are important additions to the available armamentarium within this research field. Further, to our knowledge no probes specific for CD4^+^ are presently being evaluated for immunotherapy assessment in the clinic. Indeed, the role of CD4^+^ T cells in the tumor microenvironment is of growing interest as these cells have been shown to be required for efficacious anti-tumor immunity and can target tumor cells in various ways (*reviewed by* Borst* et. al.* 8). However, the presence of several different subtypes of CD4^+^ T cells with opposing actions on anti-tumor activity in the tumor microenvironment has confounded the efforts to specifically induce CD4^+^ T cell responses for cancer immunotherapy. Tumor-specific CD4^+^ T cells in the tumor microenvironment can be reprogrammed into regulatory CD4^+^ T cells (T_regs_) that counteract cytotoxic T cell activity and are generally considered to contribute to an immuno-suppressive tumor microenvironment. The proportion of CD4^+^ T_regs_ to total CD4^+^ in the tumor microenvironment of syngeneic tumors however, has been reported to ~ 0.5-8% 54,56. Further, it should be noted that CD4^+^ T_reg_ infiltration and accumulation can correlate with a positive prognosis in certain malignancies, including colorectal, gastric and NSCLC cancer 67-69. Also, preclinical and clinical studies have shown that CD4^+^ T cells can induce durable immune-mediated tumor control, and in some cases to an even larger extent than CD8^+^ T cells 7,70-72. Together, these findings place CD4^+^ T cells as a highly relevant actor in tumor immunity and thus a potential, central prognostic marker for outcome of cancer immunotherapy. As opposed to the sole purpose of CD8^+^ T cells at the tumor site, the multifaceted and wide-ranging role of CD4^+^ T cells potentially makes CD4^+^ a better target for following T cell activity by PET imaging. By exploiting humane antibodies, the F(ab)'2 imaging approach described here can easily be modified into clinical use warranting the further development of a human version of ^89^Zr-DFO-CD4.

The immune frame-work in and around tumors is complex, making it difficult to identify broad biomarkers embracing a wide range of patients. The presence of many additional immune subsets, such as myeloid-derived suppressor cells and tumor-associated macrophages, in the periphery and the tumor microenvironment has been suggested to be a key denominator for the outcome of immunotherapy 73. Imaging markers directly reflecting the functional status, i.e. activation or exhaustion state of specific cells, have the potential to be more predictive 74 and PET imaging of activated T cells has successfully been pursued 75,76 in addition to the secreted activation markers Granzyme B 77 and IFNγ 78. Indeed, it can be debated whether a specific cellular subtype and/or activation state or the simple presence of immune cells in the tumor microenvironment is the paramount biomarker of immunotherapeutic response. Nevertheless, the PET signal from low abundance cell populations might not be sensitive enough to detect changes in response and imaging of CD4^+^ or CD8a^+^ TILs might be a useful surrogate. Future studies with a radioisotope with a shorter half-life, such as ^64^Cu (t_1/2_=12.7 hours) will enable multiple scans during and after treatment and ease the interpretation of data since the baseline T cell infiltration can vary widely, as demonstrated in this study.

## Conclusions

In the present study, we developed and validated the specificity of PET imaging radiotracers for whole-body detection and assessment of CD4^+^ and CD8a^+^ status in preclinical mouse models. These radiotracers are promising candidates as powerful, non-invasive predictors of response during the course of therapy, as they can be used to phenotype tumors at an early stage and yet allow following the treatment response within the same animal at multiple timepoints. To the best of our knowledge, this is the first report demonstrating predictivity of tumor growth response and survival using a CD4^+^-targeting PET tracer. Furthermore, it is the first study linking individual baseline CD4^+^ or CD8a^+^ levels as visualized by PET to overall response to immune checkpoint inhibition. The murine F(ab)'2 imaging approach described here can easily be modified into clinical use with human antibodies and the potential extends beyond oncology with probable value in any CD4^+^ or CD8a^+^ driven indication.

## Supplementary Material

Supplementary figures and tables.

## Figures and Tables

**Table 1:** Specifications of tracers

	89Zr-DFO-CD4	89Zr-DFO-CD8a	89Zr-DFO-IgG2b
Radiochemical yield (MBq)	11.9 ± 2.6	17.5 ± 4.3	37
Purity (%)	>99	>99	>99
Specific activity (MBq/mg)	218.7 ± 42.3	200.5 ± 39.5	553.7
Immunoreactivity (%)	90.5 ± 0.6	86.7 ± 0.7	-
Activity injected* (MBq)	0.8 ± 0.03 [0.4-1.1]	0.5 ± 0.02 [0.3-0.7]	2.4 ± 0.4 [2.2-2.6]
Protein dose injected* (µg)	2.1 ± 0.1	1.8 ± 0.3	3.3 ± 0.1

Values are mean ± SEM. *Doses listed for ^89^Zr-DFO-CD4/CD8a are from the efficacy study.

**Table 2:** Summarized IHC, flow cytometry, PET imaging and efficacy data.

			^89^Zr-DFO-CD4				^89^Zr-DFO-CD8a					
Model	CD4^+^ IHC (score)	CD4^+^ flow (%)	Tumor (max %ID/g)	Tumor / heart ratio (max)	CD8a^+^ IHC (score)	CD8a^+^ flow (%)	Tumor (max %ID/g)	Tumor / heart ratio (max)	Immune phenotype	Mean response to Sym021	Responders / non-responders CD4^+^ PET (N)	Responders / non-responders CD8a^+^ PET (N)
B16F10	0.7 ± 0.3	0.30 ± 0.10	10.3 ± 0.7	5.28 ± 0.68	1.0 ± 0.0	0.37 ± 0.19	9.7 ± 0.9	4.6 ± 0.67	Cold	No response	0/5	0/5
P815	2.0 ± 0.3	0.39 ± 0.05	14.9 ± 0.7	6.61 ± 0.96	1.0 ± 0.0	0.39 ± 0.05	17.9 ± 0.4	5.15 ± 0.38	Cold	Partial response	2/3	0/5
CT26	1.3 ± 0.2	1.14 ± 0.31	7.5 ± 0.3	8.15 ± 0.44	1.5 ± 0.0	2.95 ± 0.73	8.9 ± 0.5	9.25 ± 0.98	Hot	Responsive	1/4	1/4
MC38	1.3 ± 0.2	1.48 ± 0.2	7.7 ± 0.4	7.62 ± 0.28	1.3 ± 0.2	1.73 ± 0.42	7.1 ± 0.5	8.25 ± 0.6	Hot	Responsive	0/5	0/5
Renca	1.5 ± 0.0	0.77 ± 0.46	9.5 ± 0.5	9.99 ± 0.3	1.5 ± 0.0	0.29 ± 0.21	11.3 ± 0.8	10.15 ± 1.09	Hot/Cold	Responsive	0/5	0/5
4T1	1.2 ± 0.2	1.49 ± 0.39	11.9 ± 0.7	9.05 ± 0.4	1.3 ± 0.2	0.83 ± 0.23	12.6 ± 0.3	8.11 ± 0.41	Hot	Responsive	0/5	0/5
Sa1N	1.0 ± 0.0	1.82 ± 0.66	15.6 ± 1.4	8.49 ± 0.44	1.8 ± 0.6	1.57 ± 0.41	15.6 ± 0.8	7.26 ± 0.4	Hot	Responsive	2/3	2/3

Values are mean ± SEM. %ID/g: % injected dose per gram tissue.

## Abbreviations

%ID/g: % injected dose per gram tissue
^89^Zr: zirconium-89
ANOVA: analysis of variance
BSA: bovine serum albumin
CD3: cluster of differentiation 3
CD4: cluster of differentiation 4
CD8a: cluster of differentiation 8 alpha
CT: computed tomography
CTLA-4: cytotoxic T-lymphocyte-associated protein 4
DFO: desferrioxamine
DMSO: dimethyl sulfoxide
EDTA: ethylenediaminetetraacetic acid
FACS: fluorescence activated cell sorting
FBS: fetal bovine serum
Fc: fragment crystallizable region
HPLC: high-performance liquid chromatography
IFNγ: interferon gamma
IgG: immunglobulin G
IHC: Immunohistochemistry
LDS: lithium dodecyl sulfate
mAb: monoclonal antibody
MACS: magnetic activating cell sorting
MBq: mega becquerel
MFI: median fluorescent intensity
NaHCO_3_: sodium bicarbonate
Na_2_CO_3_: sodium carbonate
SDS: sodium dodecyl sulfate
PBS: phosphate buffered saline
PD-1: programmed cell death protein 1
PD-L1: programmed death-ligand 1
PET: positron emission tomography
SCN-Bn-DFO: *p*-SCN-Bn-Desferrioxamine
PS: penicillin-streptomycin
radio-TLC: radio-thin-layer chromatography
ROIs: region of interests
SEM: standard error of mean
TGI: tumor growth inhibition
TILs: tumor-infiltrating lymphocytes
TLC: thin-layer chromatography
XRT: external radiation therapy
