# Supplementary material for: CD4+ and CD8a+ PET imaging predicts response to novel PD-1 checkpoint inhibitor: studies of Sym021 in syngeneic mouse cancer models
Source: Theranostics. 2019 Oct 18;9(26):8221–38. doi: 10.7150/thno.37513 (PMC6857046; doi:10.7150/thno.37513)
Supplement: Supplementary file 1 — Supplementary figures and tables. [file thnov09p8221s1.pdf]

Supplementary

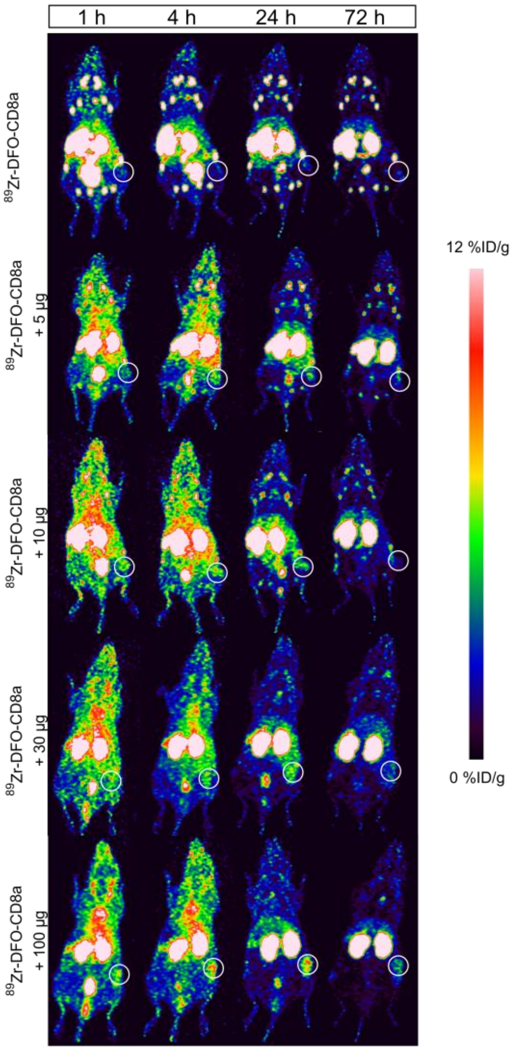

Figure S1: Optimization of dose and imaging time-point in CT26 tumor-bearing mice. Representative coronal maximum intensity projection (MIP) PET images of mice with CT26 tumors injected with ^{89}Zr-DFO-CD8a and 0, 5, 10, 30 or 100 µg CD8a-F(ab)'2. PET images were obtained 1, 4, 24 and 72 hours post-injection. Co-injection with CD8a-F(ab)'2 decreased the accumulation in lymphoid organs and increased the accumulation in tumor with increasing dose. Highest target-to-background uptake was observed with 30 µg co-injection 24 hours post-injection and was chosen as optimal imaging parameters. The PET acquisition time was 300 seconds. White circles designate the tumor. %ID/g: % injected dose per gram tissue.

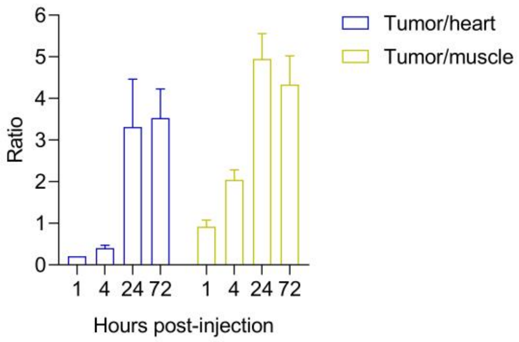

Figure S2: PET based tumor-to-background ratios in CT26 tumor-bearing mice. Tumor-to-heart and tumor-to-muscle ratios at the 1, 4, 24 and 72 hour time-point with 30 µg co-dose based on the mean PET tumor uptake of ^{89}Zr-DFO-CD8a (%ID/g) (N=3). No increase in image contrast was observed beyond 24 hours. Data are presented as mean ± SEM.

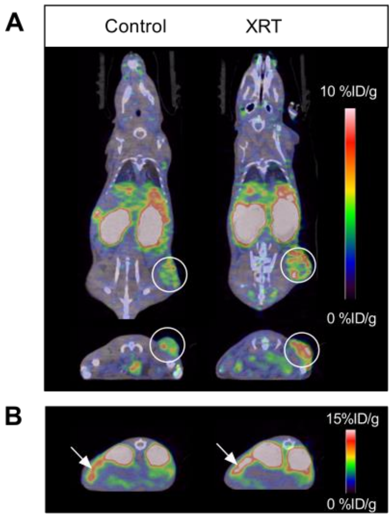

Figure S3: Effect of external radiation therapy on ^{89}Zr-DFO-CD8a uptake in CT26 tumor-bearing mice. Representative PET/CT images of the (A) tumor and (B) spleen of a control and a fractionated external radiation therapy (3x2Gy) treated CT26 tumor-bearing mouse 24 hours post-injection of ^{89}Zr-DFO-CD8a. The PET acquisition time was 300 seconds. White circles designate the tumor and white arrows designate the spleen. XRT: external radiation therapy; %ID/g: % injected dose per gram tissue.

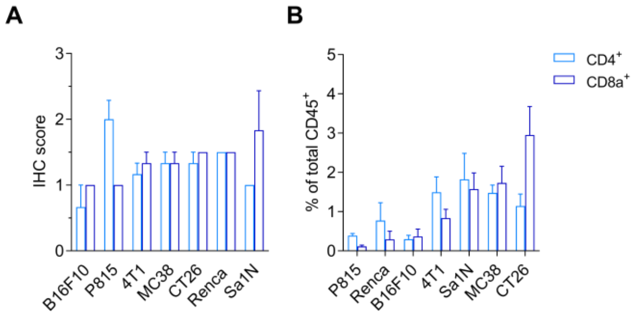

Figure S4: CD4$^+$ and CD8$^+$ subsets in syngeneic mouse models quantified by *ex vivo* methods. (A) Immunohistochemical (IHC) score of CD4$^+$ and CD8a$^+$ in cryosections of syngeneic tumor models (N=3/model). (B) Flow cytometric analysis of CD4$^+$ and CD8a$^+$ populations expressed as percentage of CD45$^+$ (N=6/model). Data are presented as mean ± SEM.

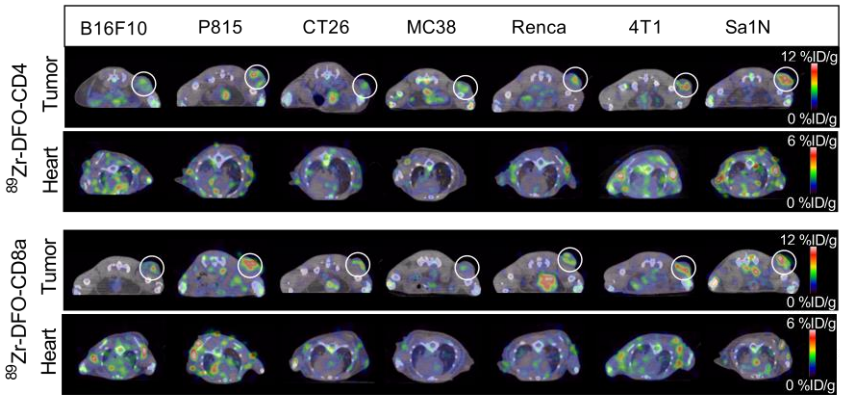

Figure S5: Representative axial PET/CT images of syngeneic tumor models. Representative PET/CT images of the tumor and heart of mice injected with ^{89}Zr-DFO-CD4 (top panel) or ^{89}Zr-DFO-CD8a (bottom panel) for each model. The PET acquisition time was 300 seconds. White circles designate the tumor. %ID/g: % injected dose per gram tissue.

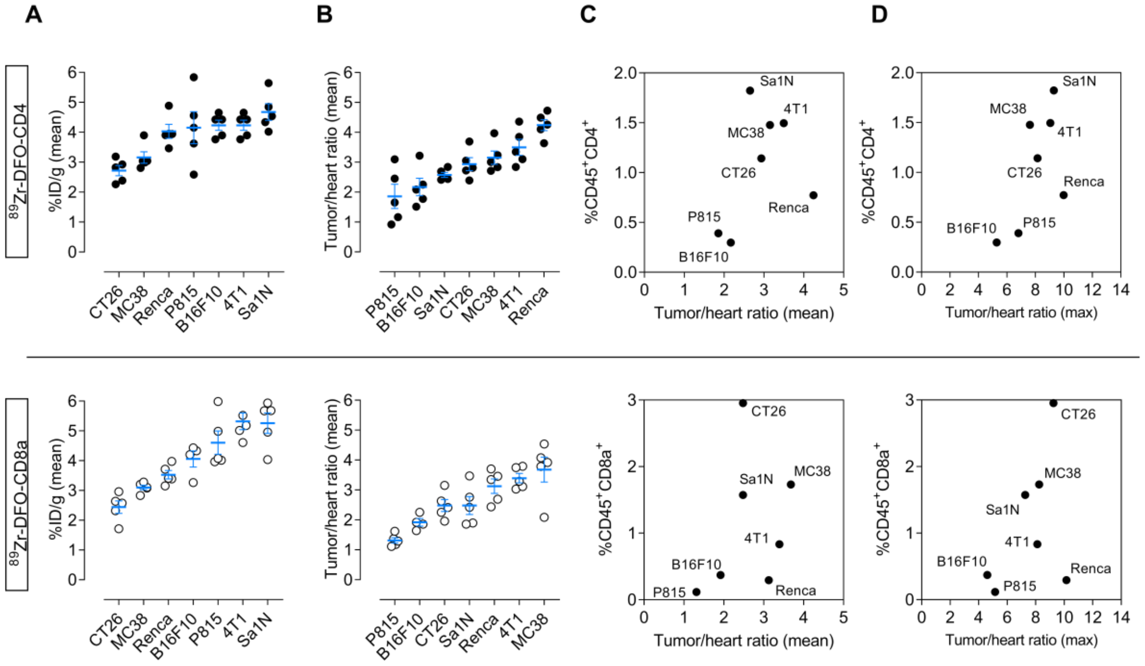

Figure S6: PET tumor uptake of ^{89}Zr-DFO-CD4 and ^{89}Zr-DFO-CD8a in syngeneic mouse models. (A) Mean ^{89}Zr-DFO-CD4 (top panel) and ^{89}Zr-DFO-CD8a (bottom panel) tumor uptake quantified from PET ROI analysis and expressed as %ID/g 24 hours post-injection of tracer in syngeneic mouse models ranked from low (left) to high (right). (B) Tumor-to-heart ratios based on the mean ^{89}Zr-DFO-CD4 (top panel) and ^{89}Zr-DFO-CD8a (bottom panel) uptake quantified from PET ROI analysis and expressed as %ID/g 24 hours post-injection of tracer ranked from low (left) to high (right). (C) Tumor-to-heart ratios based on the mean ^{89}Zr-DFO-CD4 (top panel) and ^{89}Zr-DFO-CD8a (bottom panel) uptake plotted against the average number of %CD45$^+$CD4$^+$ and %CD45$^+$CD8a$^+$ expressing cells, respectively, analyzed by flow cytometry. (D) Tumor-to-heart ratios based on the maximum ^{89}Zr-DFO-CD4 (top panel) and ^{89}Zr-DFO-CD8a (bottom panel) uptake plotted against the average number of %CD45$^+$CD4$^+$ and %CD45$^+$CD8a$^+$ expressing cells, respectively, analyzed by flow cytometry. Data are presented as mean ± SEM. %ID/g: % injected dose per gram tissue.

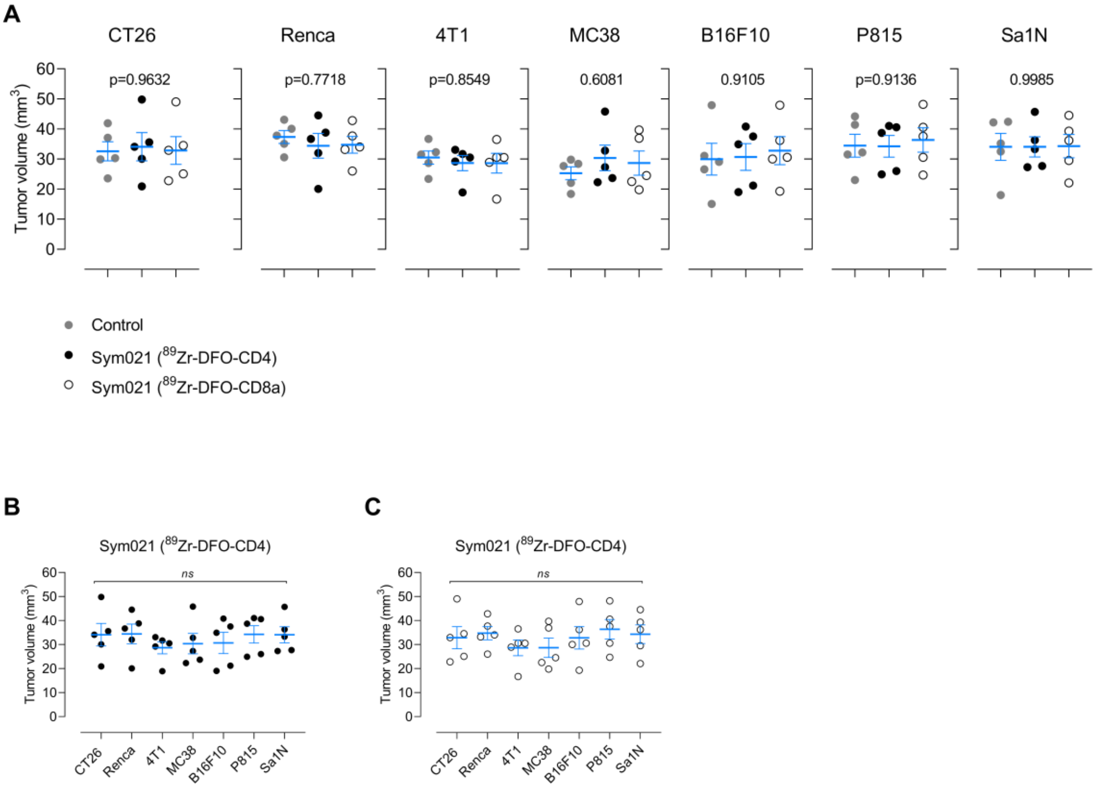

Figure S7: Tumor volumes of study groups at day of randomization. (A) Groups within each tumor model had equal tumor volume at the day of randomization (day -1 relative to start of therapy) (N=5/group). (B) All mice in the Sym021 (^{89}Zr-DFO-CD4) group had equal tumor volumes across models (N=5/model). (C) All mice in the Sym021 (^{89}Zr-DFO-CD8a) group had equal tumor volumes across models (N=5/model). Data are presented as mean ± SEM. *ns: no significance*.

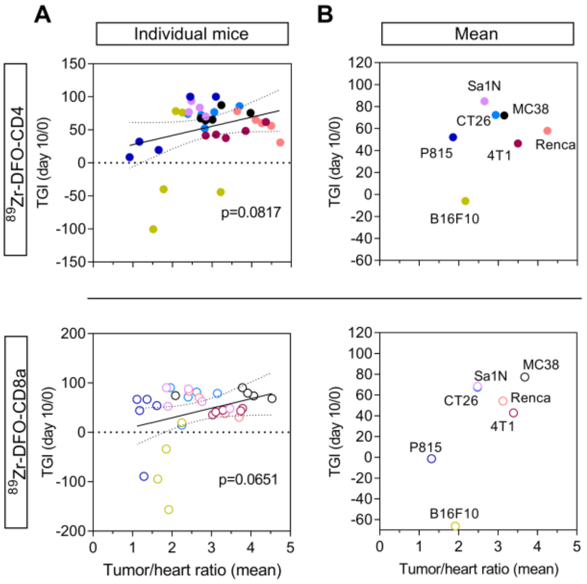

Figure S8: The tumor growth inhibition (TGI) from day 0 to day 10 in Sym021 (10 mg/kg) treated mice relative to the growth of the control group plotted against the mean ^{89}Zr-DFO-CD4 (top panel) and ^{89}Zr-DFO-CD8a (bottom panel) tumor-to-heart ratio in (A) individual mice and (B) as a mean of all tumor models (N=35/tracer, N=5/model). Data are presented as mean ± SEM.

Table S1: Tumor uptake and tumor-to-background ratios with increasing dose in CT26 tumor-bearing mice 72 hours post-injection of ^{89}Zr-DFO-CD8a (N=3/dose).

	**^{89}Zr-DFO-CD8a**		
	**Tumor uptake (%ID/g)**	**Tumor-to-muscle**	**Tumor-to-blood**
+ 0 µg	1.28 ± 0.17	3.92 ± 0.68	10.74 ± 0.91
+ 5 µg	1.97 ± 0.09	4.3 ± 0.42	12.81 ± 0.55
+ 10 µg	2.06 ± 0.08	5.67 ± 1.13	14.1 ± 2.75
+ 30 µg	2.44 ± 0.59	7.37 ± 0.68	14.69 ± 4.2
+ 100 µg	2.45 ± 0.28	5.3 ± 1.39	15.63 ± 0.89

Values are derived from gamma counting and presented as mean ± SEM. %ID/g: % injected dose per gram tissue.
